# UAV-based monitoring of fruit-manifested abiotic stress in crops under label scarcity: a case study of blossom-end rot in processing tomatoes

**DOI:** 10.3389/fpls.2026.1882618

**Published:** 2026-07-22

**Authors:** Shun Chen, Junqing Li, Yu Wang, Shunmin Li, Jianhui Jiang, Yixiang Cui, Xuejian Deng, Lulu Ma, Ze Zhang, Qiang Zhang, Xin Lv

**Affiliations:** 1 Xinjiang Production and Construction Crops Oasis Eco-Agriculture Key Laboratory, Shihezi University College of Agriculture, Shihezi, China; 2 Xinjiang Guannong Digital Agricultural Science & Technology Co., Ltd., Tiemenguan, China

**Keywords:** abiotic stress monitoring, canopy-fruit synergy, label scarcity, multispectral imagery, semi-supervised learning

## Abstract

**Introduction:**

Safeguarding the yield and quality of field crops against abiotic stresses is critical for large-scale agricultural production and precision agronomy. This study addresses the challenges of detecting concealed fruit stress and overcoming label scarcity in unmanned aerial vehicle (UAV) multispectral monitoring, using blossom-end rot (BER) in processing tomatoes as a case study.

**Methods:**

We developed a leaf–fruit synergistic Roll and Color Disease Index (RCDI), integrating fruit incidence with visible canopy phenotypic features to characterize the combined canopy–fruit stress status associated with BER. A three-level screening strategy was used to identify the optimal spectral feature set. A Multi-model Collaborative Cyclic Self-Training (MCC-ST) framework was subsequently developed to address the limited availability of severity-labeled samples.

**Results:**

The combination of GRVI, NDVI, and SAVI was identified as the optimal spectral feature set, achieving stable within-dataset binary classification performance of approximately 97% in repeated cross-validation. Under 30 random-seed repeated stratified three-fold cross-validations, MCC-ST + DT and MCC-ST + RF achieved RCDI-based BER severity-grading accuracies of 85.00% ± 0.31% and 85.07% ± 0.42%, respectively. Compared with the corresponding original DT and RF models, MCC-ST improved repeated-validation accuracy by 16.19–4.09 percentage points.

**Discussion:**

The RCDI helps bridge the observational gap between canopy signals and concealed fruit stress, while MCC-ST alleviates the bottleneck associated with label scarcity. The proposed approach provides a promising framework for crop abiotic-stress monitoring under limited-label conditions.

## 1 Introduction

Safeguarding the yield and quality of field crops against abiotic stresses is critical for precision agronomy and global food security. During the critical periods of fruit expansion and maturation, crops are highly susceptible to abiotic stresses, which can lead to severe physiological disorders, fruit abscission, or quality degradation. For instance, in the context of processing tomatoes, blossom-end rot (BER) is an abiotic physiological disorder triggered by localized calcium deficiency and water imbalance, rather than a pathogen-induced disease (Sethi et al., 2024). In severe scenarios, this disorder can cause catastrophic yield losses ranging from 30% to 100% in large-scale production, severely undermining the economic viability of the agricultural system. Timely detection of such abiotic stresses followed by targeted variable-rate management is a critical approach to mitigating yield losses.

Historically, the monitoring of abiotic stress in large-scale agricultural production has predominantly relied on manual field surveys, which are inherently time-consuming, labor-intensive, and prone to subjective errors. With the advancement of unmanned aerial vehicle (UAV) technologies, multispectral remote sensing has emerged as the prevailing methodology for the rapid, large-scale, and non-destructive monitoring of crop health status. Utilizing UAV RGB imagery and deep learning methods, deep networks trained on combinations of different color spaces and vegetation indices achieved an accuracy of 95.80% in detecting Esca disease in vineyards (Kerkech et al., 2018). The integration of UAVs with multispectral remote sensing technology to train machine learning models has also accurately identified wheat rust and blight (Joshi et al., 2024). Furthermore, models trained using UAV-acquired RGB and vegetation index images can significantly evaluate the canopy severity of wheat leaf rust (Bhandari et al., 2020). The aforementioned studies demonstrate that modeling by integrating UAV remote sensing data with ground disease indices can accurately monitor the occurrence and severity of diseases.

Blossom-end rot manifests exclusively on the tomato fruit. Traditionally, the disease index is calculated based on the ratio of symptomatic fruits to the total fruit count. However, unmanned aerial vehicles (UAVs) are incapable of directly monitoring the fruits; instead, they must infer the disease status utilizing canopy spectral information. Under identical conditions, the severity of abiotic stress exhibited at the fruit level frequently diverges from the severity manifested at the canopy leaf level. Therefore, it is insufficient to construct an assessment model solely based on the correlation between canopy spectra and the fruit disease index. Prescription maps generated from such a model may also lack reliability and comprehensiveness for guiding supplementary fertilization. Studies have indicated that conventional deficit irrigation significantly increases BER incidence compared to alternate partial root-zone irrigation, suggesting that water deficit is a primary environmental trigger for BER (Millones-Chanamé et al., 2019). Furthermore, physiological leaf rolling is a distinct morphological manifestation of crops under water stress (O’Toole and Cruz, 1980); thus, physiological leaf rolling in processing tomatoes can serve as a critical early warning symptom of BER. Research has also demonstrated a significant negative correlation between BER incidence and leaf calcium content, where lower calcium levels in plant leaves correspond to a higher proportion of BER-symptomatic fruits (Wu et al., 2025). During calcium deficiency, the chlorophyll content in tomato leaves decreases significantly (Rangnekar, 1975), causing the upper leaves to turn brown. Consequently, leaf color serves as a vital feature for identifying physiological calcium deficiency. Monitoring the physiological status of leaves, therefore, essentially constitutes monitoring the environmental triggers of water stress and the physiological precursors of insufficient calcium absorption that induce BER. In certain diagnostic frameworks, Kerala Agricultural University has begun employing color variations in emerging leaves and upward leaf rolling to assess calcium deficiency and BER status. In summary, constructing a disease index that integrates both canopy leaves and fruits—utilizing leaf status to reflect disease stress and fruit status to reflect the actual disease manifestation—is both necessary and justifiable to establish a robust link between UAV-acquired canopy remote sensing data and ground-based disease indices.

The prerequisite for precise monitoring via UAV remote sensing is the acquisition of large volumes of remote sensing data and corresponding ground-truth data to train high-precision models. However, the high planting density of processing tomatoes and the intertwining of plants make ground-truth data collection both difficult and time-consuming, particularly when attempting to ensure data accuracy while avoiding crop damage. This often leads to a temporal misalignment between remote sensing data and ground-truth data, resulting in a reduction in model accuracy. Therefore, during data acquisition, the volume of ground-truth data corresponding to remote sensing data is typically limited. Training machine learning models directly with a small number of labeled samples is highly susceptible to overfitting (Charilaou and Battat, 2022). Semi-supervised learning theory offers a solution to this bottleneck, as its core principle involves jointly training models using a limited set of labeled data alongside a large volume of unlabeled data (Yang et al., 2023). By converting large-scale unlabeled data into pseudo-labeled data to train classification models, model accuracy can be significantly enhanced. Nevertheless, current semi-supervised learning methods entail the risk of confirmation bias. If an initial model assigns incorrect pseudo-labels with high confidence, these errors can cause the model to deviate or even collapse (Ding et al., 2025).

To address the dual challenges of physiological complexity and methodological limitations, this study constructed a disease index that integrates leaf morphology and color, namely the Roll and Color Disease Index (RCDI). We also designed a Multi-model Cooperative Cyclic Self-Training (MCC-ST) framework. This framework leverages extensive canopy remote sensing data obtained via UAVs alongside limited ground-truth data to perform high-confidence pseudo-labeling on large-scale unlabeled datasets, thereby improving the model’s accuracy in assessing BER severity. By coupling a leaf–fruit synergistic RCDI-based BER severity index with UAV canopy remote sensing data, this research aims to evaluate RCDI-based BER severity, which represents an integrated canopy–fruit stress status rather than a fruit-only BER incidence endpoint, offering a universal technical approach for monitoring fruit-manifested abiotic physiological disorders, particularly in scenarios where large-scale ground-measured data are unavailable.

## 2 Materials and methods

### 2.1 Experimental design

**Figure 1:**
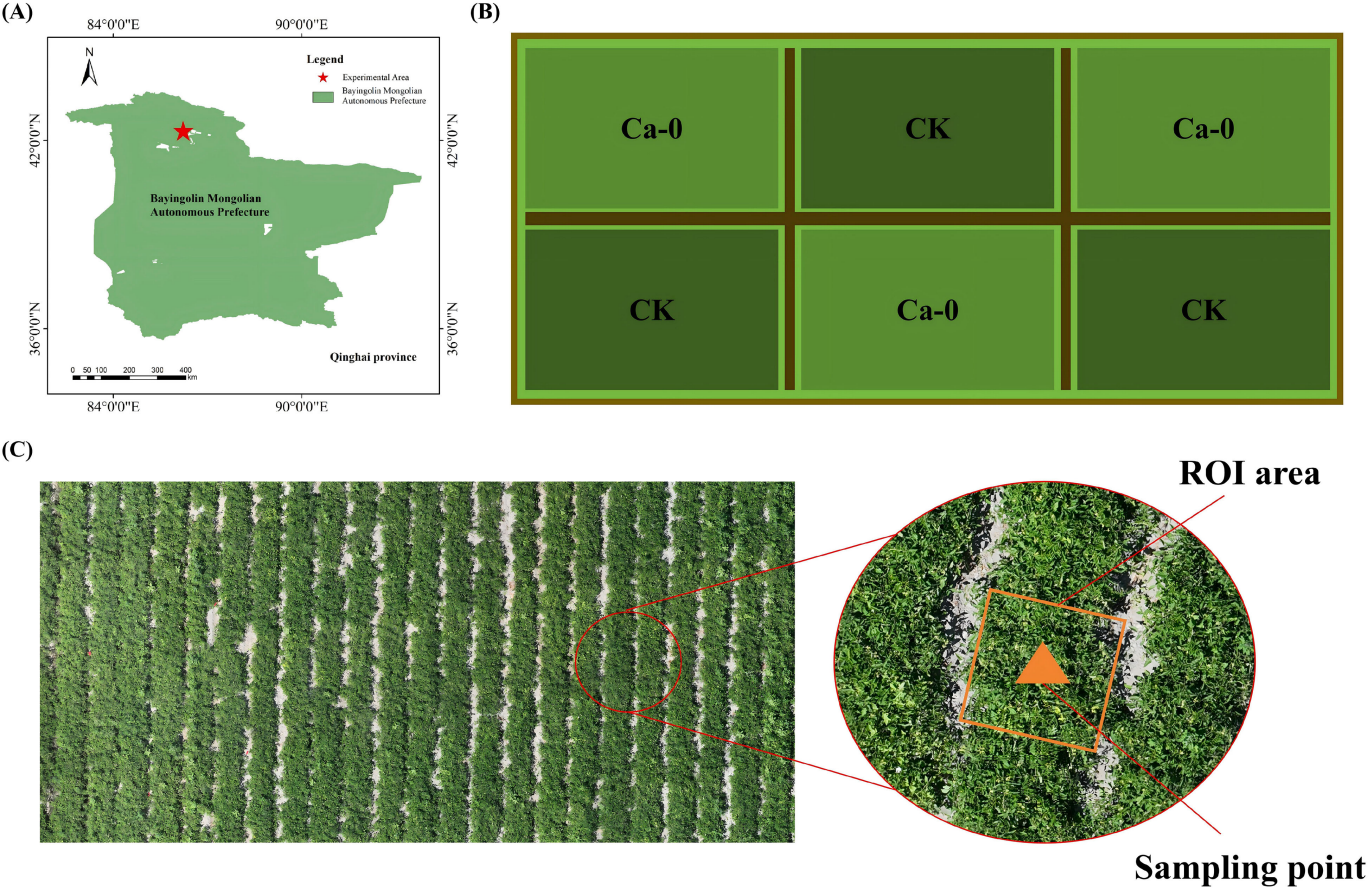
Location and overview of the experimental area. **(A)** Geographic location of the experimental site in the 22nd Regiment of Tiemenguan City, Xinjiang, China. **(B)** Layout of the experimental plots used for processing tomato BER monitoring. **(C)** UAV orthomosaic showing the representative field rows, ROI extraction area, and corresponding sampling point.

The experimental site of this study was located in the 22nd Regiment of Tiemenguan City, Second Division of the Xinjiang Production and Construction Corps. The soil type is characterized as loam with medium fertility, providing a suitable environment for tomato growth. The experimental variety selected was ‘Jinfan 1015’, a cultivar widely planted in the region known for its vigorous growth, high fruit firmness, and pronounced sensitivity to calcium. The experimental area was organized into two treatments, each with three random replicates: namely the control group (CK) and the calcium-water stress group (Ca-0), with three replicates for each treatment. The six experimental plots were arranged in a randomized field layout to reduce the influence of spatial heterogeneity within the field. Each plot had an area of approximately 15 m × 30 m, 450 m², and adjacent plots were separated by 2 m buffer zones to minimize interference caused by irrigation, fertilization, and edge effects. The control group (CK) received sufficient water-soluble calcium and magnesium fertilizers throughout the entire growth cycle via an integrated water and fertilizer system. This treatment strictly followed the nutrient requirements for the target yield of processing tomatoes. The stress group (Ca-0) involved the complete cessation of water supply from the full-bloom stage to the fruit expansion stage. The geographic location and a general overview of the experimental site are presented in **Figure 1**.

The plants in the experimental plot were transplanted on April 8, 2024, utilizing a standardized cultivation technique characterized by plastic film mulching and drip irrigation. Specifically, a ‘one film, two tubes, and two rows’ configuration was adopted, featuring a film width of 120 cm, a row spacing of 35 cm, and a plant spacing of 30 cm, resulting in a theoretical planting density of 2,850 plants per mu. A total of 10 irrigation events were conducted throughout the entire growth cycle, with an irrigation interval ranging from 8 to 10 days per application.

### 2.2 Construction of the RCDI-based BER severity index

This study used leaf status to reflect disease-related stress and fruit status to represent the actual disease manifestation. Based on this principle, we constructed a leaf-fruit synergistic disease index, namely the Roll and Color Disease Index (RCDI), to evaluate the incidence trend and severity of blossom-end rot (BER) in processing tomatoes. The calculation formula is shown in Equation 1.

(1) $$RCDI=\;\frac{N_{disease}}{N_{total}}\times W_{1}+L_{R}\times W_{2}+L_{C}\times W_{3}$$

In practical surveys and calculations at each sampling point, Ndiseased represents the number of BER-symptomatic fruits in the survey, Ntotal represents the total number of surveyed fruits, L_R_ represents the influence mapping value of the leaf rolling degree of functional leaves, and L_C_ represents the influence mapping value of the leaf color condition of emerging leaves; specific details are presented in **Table 1**. In this study, all components in Equation 1 were expressed on a normalized 0–1 scale. Specifically, (Ndisease/Ntotal) represents the fruit-incidence component and ranges from 0 to 1. L_R_ and L_C_ represent the normalized influence mapping values of functional leaf rolling and emerging leaf color, respectively, and both range from 0 to 1 according to **Table 1**. Since the three weights sum to 1, the final RCDI also ranges from 0 to 1. Therefore, the RCDI thresholds in **Table 2** were expressed using the same 0–1 scale. W1, W2, and W3 are the weight parameters. In this study, the weights of RCDI were determined using the Analytic Hierarchy Process (AHP) combined with the physiological pathogenesis of Blossom-End Rot in processing tomatoes. The AHP-derived weights were used as physiologically constrained prior weights rather than globally optimized data-driven weights. Fruits were assigned the highest importance because fruit symptoms are the most direct criterion for identifying BER. Emerging leaf color reflects tissue necrosis and calcium deficiency. Since calcium deficiency is the direct endogenous cause of BER, L_C_ was assigned an intermediate weight. Functional leaf rolling reflects water stress. Although water deficit can trigger BER, its effect is considered an indirect environmental factor; therefore, L_R_ was assigned the lowest weight. Therefore, the importance of L_R_ is the lowest. Based on the aforementioned physiological logic, this study constructed a judgment matrix and assigned values using the scaling method: fruits are moderately more important than L_C_ (Scale = 2); fruits are significantly more important than L_R_ (Scale = 3); and L_C_ is slightly more important than L_R_ (Scale = 1.5). By normalizing the matrix and calculating the maximum eigenvector, the weight vector *W* = [0.539, 0.164, 0.297] was obtained; thus, the weights are *W_1_* = 0.539, *W_2_* = 0.164, and *W_3_* = 0.297. It should be noted that RCDI was not designed as a fruit-only BER incidence endpoint. Instead, it was designed as an integrated canopy–fruit severity target that combines fruit manifestation with canopy stress symptoms. Therefore, models trained with RCDI labels should be interpreted as predicting RCDI-based BER severity rather than fruit-only BER incidence.

**Table 1:** Specific meanings and numerical values of *L_R_* and *L_C._*.

Level	L_R_ (Reflecting water stress and calcium transport resistance)	L_C_ (Reflects Ca deficiency and necrosis levels)	Influence mapping value
Level 0	Leaves are fully expanded with normal color and no rolling.	Normal green leaves with smooth margins and no yellowing or necrosis.	0
Level 1	Leaf margins are slightly curled up, appearing in a shallow rolling shape.	Slightly dull or pale leaves with mild marginal yellowing and no necrosis.	0.2
Level 2	Distinct upward rolling, but margins are not in contact.	Visible yellowing of leaf margins or young leaves, with a few small brown spots.	0.4
Level 3	Rolled into a tube shape with margins close to each other; texture becomes hardened.	Pronounced yellowing with scattered brown spots or limited marginal scorching.	0.6
Level 4	Severely rolled with margins contacting or overlapping; texture becomes brittle.	Obvious marginal browning or scorching extending inward, with partial shriveling.	0.8
Level 5	Rolled into a spiral shape; veins are twisted and extremely brittle.	Severe chlorosis, scorching, or necrosis, with large necrotic areas or partial leaf death.	1

**Table 2:** Mapping of RCDI ranges to BER severity levels.

RCDI range	Severity level	Physiological status interpretation
0-0.03	Healthy (Level 0)	Plant water balance is maintained, calcium transport is unobstructed, and fruits exhibit no lesions.
0.03–0.10	Potential risk (Level 1)	Fruits have not yet or rarely developed symptoms, but functional leaves exhibit slight rolling or emerging leaves show chlorosis.
0.10-0.25	Mild occurrence (Level 2)	Sporadic fruits exhibit BER symptoms, and leaf rolling is prominent.
0.25-0.45	Moderate occurrence (Level 3)	Diseased fruit rate increases; functional leaves appear cup-shaped or tube-shaped.
0.45-0.7	Severe (Level 4)	A large number of fruits exhibit BER, and margins of emerging leaves show necrosis and scorching.
> 0.70	Extremely severe (Level 5)	More than half of the fruits are rotten; leaves are severely rolled and scorched, and the growing point is necrotic.

RCDI was expressed on a 0–1 scale for severity grading. Because no official or industry-standard severity classification is available for the newly proposed RCDI, the thresholds were defined by the authors based on general plant disease severity assessment principles, the biological meaning of the RCDI components, and field-observed BER symptom progression. The grading system was used to provide reproducible ordinal labels for model training and severity evaluation.

**Figure 2:**
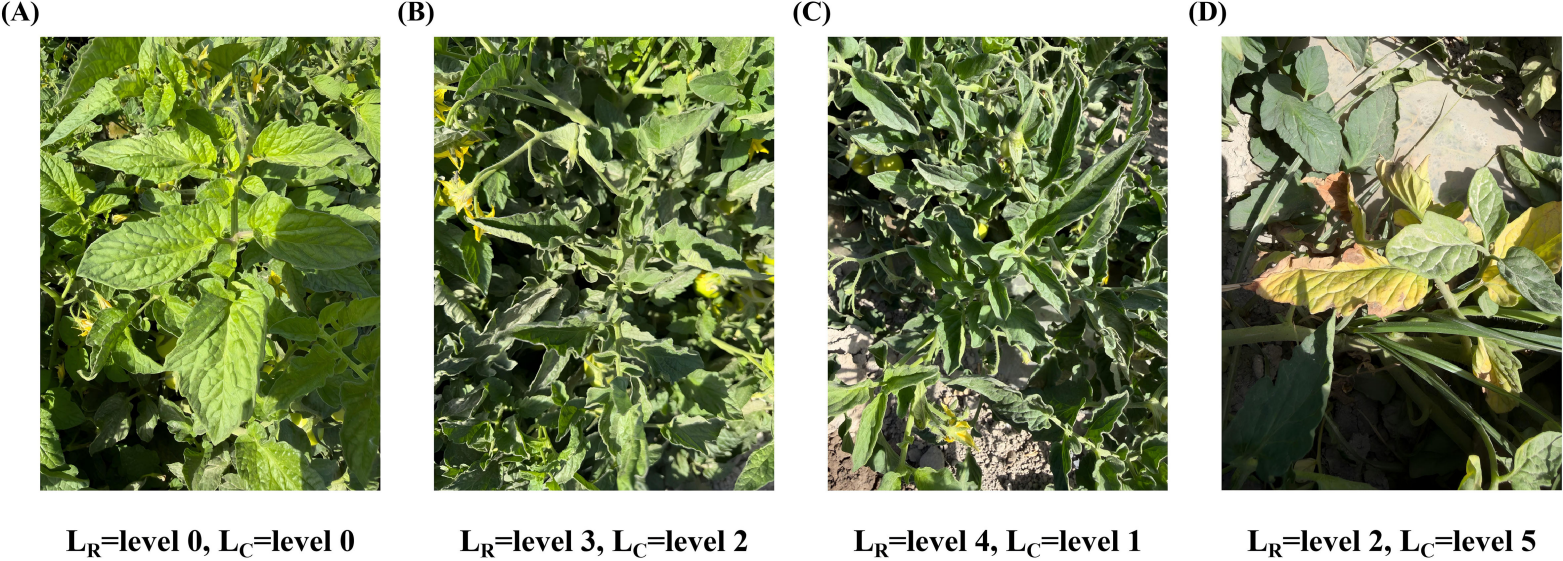
Representative field images of leaf color (L_C_) and leaf rolling (L_R_) scoring levels used for RCDI-based BER severity assessment. **(A)** L_R_ Level 0 and L_C_ Level 0; **(B)** L_R_ Level 3 and L_C_ Level 2; **(C)** L_R_ Level 4 and L_C_ Level 1; and **(D)** L_R_ Level 2 and LC Level 5. LR and LC were scored independently; therefore, the two scores of the same sampling point were not necessarily identical.

Representative field images showing typical L_C_ and L_R_ scoring combinations are provided in **Figure 2** to understand the visual criteria used for RCDI-based BER severity assessment.

Due to the incorporation of leaf phenotypic indicators, the numerical connotation of RCDI is more comprehensive than that of conventional fruit-incidence-based disease incidence. Even if extensive fruit rot has not yet occurred, high L_R_ or L_C_ scores can increase the RCDI value, thereby providing an early warning function.

Because RCDI is a newly constructed leaf-fruit synergistic index, no official or industry-standard severity classification system is currently available for this specific index. Therefore, the RCDI severity grading system used in this study was defined by the authors. The threshold setting was based on three considerations: (1) the general practice in plant disease assessment of transforming continuous or percentage-based disease measurements into ordinal severity classes; (2) the biological meaning of the three RCDI components, namely fruit incidence, functional leaf rolling (L_R_), and emerging leaf color (L_C_); and (3) the field-observed progression of BER symptoms from asymptomatic or potential-risk status to severe fruit rot, leaf scorching, and growing-point necrosis.

For consistency with conventional percentage-based disease assessment, the weighted RCDI value was expressed on a 0–1 scale for severity grading. The threshold of 0.03 was used to separate healthy plants from potential-risk plants, allowing a small tolerance for trace symptoms and field scoring uncertainty. The 0.03–0.1 interval was defined as the potential-risk stage, in which fruit symptoms were absent or rare but early canopy stress symptoms, such as slight functional leaf rolling or emerging-leaf chlorosis, could already be observed. The 0.1–0.25 interval was defined as mild occurrence, representing sporadic BER symptoms and clear canopy warning signals. The 0.25–0.45 interval was defined as moderate occurrence, indicating an increased diseased fruit rate and more obvious leaf rolling. The 0.45–0.7 interval was defined as severe occurrence, corresponding to widespread fruit symptoms and leaf-margin necrosis. Values above 0.7 were classified as extremely severe, indicating that more than half of the fruits were affected and that severe canopy rolling, scorching, or growing-point necrosis was present. The complete RCDI severity grading criteria are shown in **Table 2**.

These thresholds were designed as operational categories on the normalized 0–1 RCDI scale. The lowest interval represents plants with almost no fruit symptoms and no obvious canopy stress, whereas the potential-risk interval represents cases in which fruit symptoms are absent or rare but slight leaf rolling or young-leaf color changes have appeared. The subsequent intervals correspond to progressively stronger fruit incidence, leaf rolling, and young-leaf color abnormality. Therefore, the thresholds were used to stratify the integrated fruit–canopy stress status rather than to represent fruit incidence alone. For example, if 10 BER-symptomatic fruits were observed among 40 surveyed fruits. If functional leaf rolling was scored as Level 2, the corresponding L_R_ mapping value would be 0.4; if emerging leaf color was scored as Level 1, the corresponding L_C_ mapping value would be 0.2. Using (W_1 = 0.539), (W_2 = 0.164), and (W_3 = 0.297), the RCDI would be calculated as follows:[RCDI = 0.25*0.539 + 0.4*0.164 + 0.2*0.297 = 0.25975]. According to **Table 2**, this sampling point would be classified as Moderate occurrence.

To further evaluate whether RCDI provides additional information beyond conventional fruit-incidence-based assessment, the conventional disease incidence index (DI = Ndiseased/Ntotal) was also calculated for the same 73 ground sampling points and used as a comparative baseline in the subsequent model validation.

To clarify the sample accounting, the samples used in this study were divided into two main groups. First, 73 ground RCDI sampling points were established with precise fruit counts, functional leaf rolling scores, emerging leaf color scores, and final RCDI severity labels. These samples were used for supervised disease grading and served as the initial severity-labeled dataset for the MCC-ST framework. Second, 550 random sampling points were established for binary healthy/BER-stressed classification. These random points were assigned binary labels according to the presence of diseased fruits and canopy symptoms, but they did not contain precise RCDI severity labels based on detailed fruit counts, L_R_, and L_C_ measurements. Therefore, in the MCC-ST framework, these 550 random points were used as the severity-unlabeled candidate pool for pseudo-label generation. The pseudo-labeled samples generated by MCC-ST were used only to augment the training set, whereas model validation for disease grading was performed using held-out samples from the 73 ground RCDI sampling points.

To provide physiological support for the inclusion of functional leaf rolling (LR) and emerging leaf color (LC) in the RCDI, plant samples corresponding to healthy and BER-stressed sampling points were collected. The measured physiological parameters included leaf water content, stem water content, leaf total calcium content, stem total calcium content, and fruit total calcium content. Normality was assessed using the Shapiro–Wilk test. Differences in physiological parameters between healthy and BER-stressed plants were tested using a two-sided independent-samples t-test.

### 2.3 Data acquisition

To construct a reliable remote sensing inversion model, it is imperative to acquire ground observation data that are strictly spatiotemporally matched with the remote sensing data. This study conducted synchronized ground surveys and UAV flights during the early fruit expansion stage (June 20) and the peak fruiting stage (July 16). These two periods correspond to the initial onset and peak incidence stages of Blossom-End Rot, respectively, thereby covering the critical dynamic processes of disease progression.

#### 2.3.1 UAV multispectral image acquisition

Multispectral images of the experimental field were acquired using a DJI Mavic 3 Multispectral (DJI, China) during the sampling periods. Image collection was conducted between 13:00 and 14:00 under clear, cloudless, and windless weather conditions. The UAV platform was equipped with 5-megapixel (MP) imaging sensors, with the lens oriented vertically downward (nadir). The camera shutter was set to 1/240 s, and images were captured at equal time intervals at an altitude of 20 m above ground level. Both the forward and side overlap were set to 75%. The acquired raw images were imported into DJI Terra for stitching; during radiometric calibration, photos of the reflectance calibration panel were imported and the corresponding reflectance values were entered to generate single-band orthomosaics. Finally, the orthomosaics were imported into ENVI 5.3 for layer stacking to obtain the complete multispectral imagery. The specific parameters of the UAV are shown in **Table 3**.

**Table 3:** UAV-related data specifications.

Parameter	Specifications
Multispectral bands	Green (G): 560nm ± 16nm; Red (R): 650nm ± 16nm; RedEdge (RE): 730nm ± 16nm; NIR: 860nm ± 26nm;
Photo format	TIFF
Shooting mode	Single shot: 500 MP Timed shot: 500 MP TIFF: 2/3/5/7/10/15/20/30/60 s
RTK positioning accuracy	RTK Fixed Solution: Horizontal:1 cm + 1 ppm Vertical: 1.5 cm + 1 ppm
Image sensor	1/2.8-inch CMOS, 5 million effective pixels (5 MP)

After radiometric correction and layer stacking, the sampling-point coordinates were overlaid on the multispectral orthomosaic to extract vegetation-index values. For each ground sampling point or random sampling point, a region of interest (ROI) was defined around the corresponding field location, and the mean values of VIs within the ROI were extracted as model inputs. No additional semantic segmentation mask was applied in this study. Instead, the potential influence of soil and plastic-mulch background was reduced by ROI-level extraction.

#### 2.3.2 Ground data acquisition

Ground data acquisition was conducted synchronously with UAV image acquisition on June 20 and July 16, corresponding to the early fruit expansion stage and the peak fruiting stage, respectively. A total of 73 ground-truth disease index sampling points and 550 random sampling points were established across the experimental field. The 73 ground-truth disease index sampling points were distributed across the CK and Ca-0 treatments and their three replicates, covering different levels of blossom-end rot severity. The sampling points were arranged within the central area of each plot, while border rows and plot edges were avoided to reduce edge effects.

At each ground-truth disease index sampling point, the three plants nearest to the recorded sampling coordinate were investigated. For these three plants, the total number of fruits and the number of BER-symptomatic fruits were recorded and summed as the fruit-level observation for that sampling point. Meanwhile, the rolling degree of functional leaves (L_R_) and the color status of emerging leaves (L_C_) were evaluated based on the overall leaf phenotype of the three investigated plants according to the criteria listed in **Table 1**. The RCDI value and the corresponding disease severity grade were then calculated for each ground-truth disease index sampling point. To reduce subjective bias in ground-truth labeling, all field investigators were trained using the same scoring criteria before sampling. When inconsistent judgments occurred, the sample was rechecked in the field and the final label was determined after discussion.

**Figure 3:**
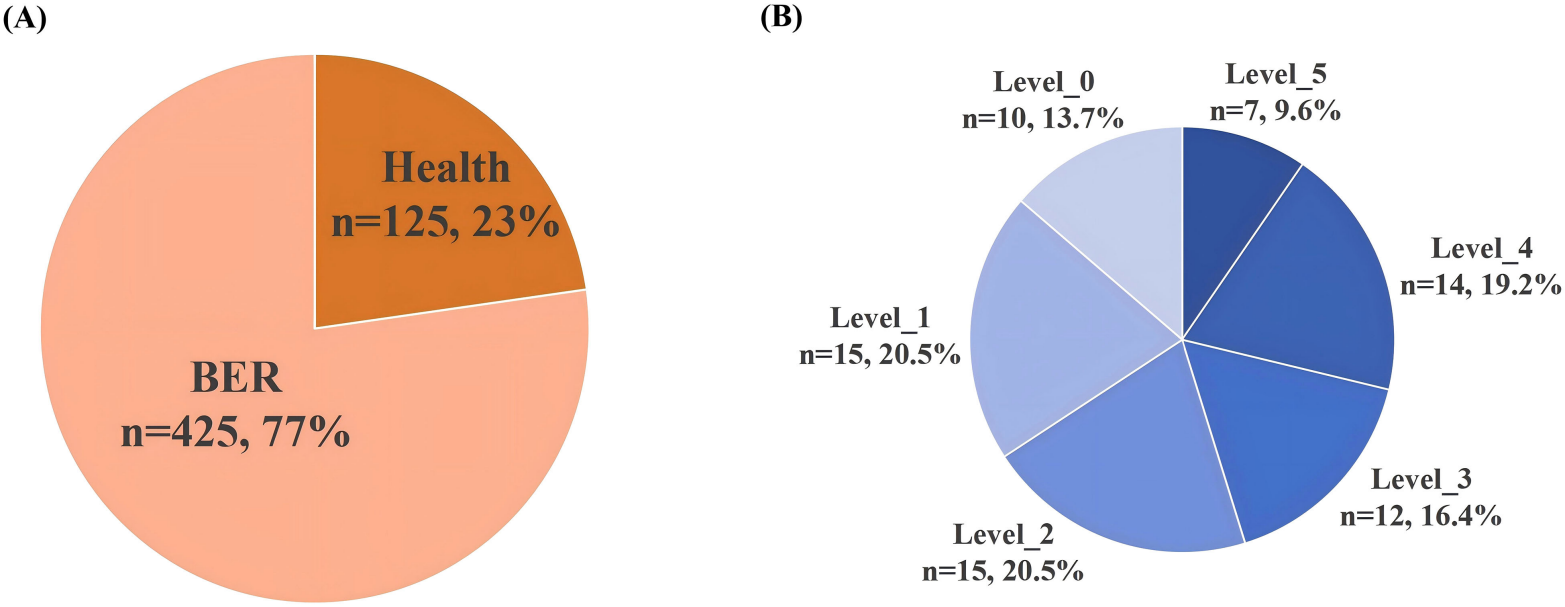
Data distribution map. **(A)** shows the distribution of normal and BER-symptomatic samples in the binary classification dataset. **(B)** shows the sample number of each RCDI-based BER severity level among the 73 labeled samples.

For the 550 random sampling points, field observations were used to determine whether each point was affected by BER stress. If BER-symptomatic fruits were observed, the point was recorded as label_disease. If no symptomatic fruit was observed, L_R_ and L_C_ were further assessed. Points with both L_R_ and L_C_ equal to 0 were recorded as healthy samples, whereas points with any non-zero L_R_ or L_C_ score were recorded as stressed samples. Therefore, the 73 ground-truth disease index sampling points were used for RCDI-based severity grading, while the 550 random sampling points were used for binary classification and pseudo-label generation. The final distribution of binary labels and RCDI severity grades is shown in **Figure 3**.

As shown in **Figure 3B**, the 73 RCDI-labeled samples were distributed across BER severity levels as follows: level_0 = 10, level_1 = 15, level_2 = 15, level_3 = 12, level_4 = 14, and level_5 = 7. This distribution provides the basis for evaluating the potential influence of class imbalance on BER severity grading.

To clarify the sample accounting, the samples used in this study were divided into two main groups. First, 73 ground RCDI sampling points were established with precise fruit counts, functional leaf rolling scores, emerging leaf color scores, and final RCDI severity labels. These samples were used for supervised disease grading and served as the initial severity-labeled dataset for the MCC-ST framework. Second, 550 random sampling points were established for binary healthy/BER-stressed classification. These random points were assigned binary labels according to the presence of diseased fruits and canopy symptoms, but they did not contain precise RCDI severity labels based on detailed fruit counts, L_R_, and L_C_ measurements. Therefore, in the MCC-ST framework, these 550 random points were used as the severity-unlabeled candidate pool for pseudo-label generation. The pseudo-labeled samples generated by MCC-ST were used only to augment the training set, whereas model validation for disease grading was performed using held-out samples from the 73 ground RCDI sampling points.

For spatial matching between ground observations and UAV imagery, the UAV multispectral orthomosaic was georeferenced using the onboard RTK module of the DJI Mavic 3 Multispectral platform. During field investigation, the geographic location of each sampling point was recorded using a smartphone-based positioning application. The recorded sampling coordinates were then imported into GIS software and overlaid onto the RTK-supported UAV multispectral orthomosaic. Considering the positioning uncertainty of smartphone-based field records, the locations of sampling points were further checked and adjusted according to plot boundaries, plant rows, field sampling records, and visible ground features in the orthomosaic. For each sampling point, the corresponding region of interest (ROI) was extracted from the UAV image using a buffer centered on the corrected sampling coordinate. Pixels obviously affected by soil background, plastic film, shadows, or field roads were excluded when extracting vegetation index values.

For each ground sampling point, a 275 × 275 pixel ROI was extracted from the orthomosaic image, centered on the corrected sampling location. This ROI size was selected to fully encompass the canopy of the three investigated tomato plants while minimizing interference from adjacent plots, bare soil, shadows, and other background elements. Vegetation indices were calculated from the canopy pixels within each ROI and subsequently used for feature extraction and machine-learning model development. To ensure the reproducibility and comparability of the extracted features, all ROIs were generated using the same extraction procedure and image scale throughout the study.

### 2.4 Feature selection and semi-supervised learning methods

#### 2.4.1 Selection of vegetation indices

Based on the multispectral bands acquired by the UAV platform, candidate vegetation indices were calculated to characterize canopy pigment status, vegetation coverage, canopy structure, and background-adjusted spectral responses. These vegetation indices were used as candidate features for subsequent feature screening and model construction. The vegetation indices calculated in this study are shown in **Table 4**.

**Table 4:** Details of vegetation indices.

Vegetation index	Formula	Reference
Normalized Difference Vegetation Index	$\text{NDVI}=\frac{\text{NIR}-\text{Red}}{\text{NIR}+\text{Red}}$	(Rouse et al., 1974)
Soil Adjusted Vegetation Index	$\text{SAVI}=\frac{\text{NIR}-\text{Red}}{\text{NIR}+\text{Red}+\text{L}}\times (1+\text{L})$	(Huete, 1988)
Optimized Soil Adjusted Vegetation Index	$\text{OSAVI}=\frac{\text{NIR}-\text{Red}}{\text{NIR}+\text{Red}+0.16}\times (1+0.16)$	(Rondeaux et al., 1996)
Normalized difference red-edge index	$\text{NDRE}=\frac{\text{NIR}-\text{RedEdge}}{\text{NIR}+\text{RedEdge}}$	(Gitelson and Merzlyak, 1994)
Chlorophyll Index - RedEdge	${\text{Cl}}_{\text{red}\_\text{edge}}=\frac{\text{NIR}}{\text{RedEdge}}-1$	(Gitelson et al., 2005)
Green-Red Vegetation Index	$\text{GRVI}=\frac{\text{Green}-\text{Red}}{\text{Green}+\text{Red}}$	(Bendig et al., 2015)
Green Normalized Difference Vegetation Index	$\text{GNDVI}=\frac{\text{NIR}-\text{Green}}{\text{NIR}+\text{Green}}$	(Gitelson and Merzlyak, 1998)
Ratio Vegetation Index	$\text{RVI}=\frac{\text{NIR}}{\text{Red}}$	(Birth and McVey, 1968)
Renormalized difference vegetation index	$\text{RDVI}=\frac{\text{NIR}-\text{Red}}{\sqrt{\text{NIR}+\text{Red}}}$	(Roujean and Breon, 1995)
Chlorophyll Index - Green	${\text{Cl}}_{\text{green}}=\frac{\text{NIR}}{\text{Green}}-1$	(Gitelson et al., 2003)

#### 2.4.2 Three-stage screening of sensitive features

Given that vegetation indices (VIs) exhibit varying sensitivities to BER in processing tomatoes and often contain high levels of redundant information, this study established a multi-stage sensitive feature screening system. The objective was to ensure that model input variables are closely associated with BER while minimizing information redundancy.

First, the overlapping rate of Kernel Density Estimation (KDE) was calculated using the Probability Density Function (PDF) to intuitively compare the distribution overlap between healthy and diseased samples within the feature space. A lower overlapping rate indicates a more pronounced distributional difference, signifying superior inter-class separability for the given VI (Koliander et al., 2022). Subsequently, point-biserial correlation analysis was utilized to quantify the linear relationship between continuous VIs and binary disease labels. Features demonstrating a sharp response to disease stress were preliminarily selected based on significance tests (Cinquanta et al., 2021). Lastly, Pearson correlation analysis was performed to identify redundant vegetation indices. Because most candidate vegetation indices were calculated from a limited number of UAV multispectral bands, some indices inevitably contained highly overlapping spectral information. In this study, (|r| > 0.9) was used as a conservative threshold to identify extremely high pairwise redundancy. This threshold was not intended to remove all moderately correlated variables, but to exclude near-duplicate indices that provided largely repetitive spectral information. For highly correlated feature pairs, indices with clearer physiological interpretation, stronger disease sensitivity, or greater robustness against soil background interference were preferentially retained (Carrasco-López et al., 2024).

It should be noted that the three-stage feature screening was used as an initial sensitive-feature identification procedure to determine a fixed and physiologically interpretable vegetation-index set. After feature set was selected, this feature set was kept unchanged in all subsequent classification and grading models. Therefore, the cross-validation results should be interpreted as the validation of this predefined feature set within the current dataset, rather than as a fully nested validation of the feature-selection procedure itself.

#### 2.4.3 Multi-model collaborative cyclic self-training

To address the practical challenge of scarce labeled samples alongside an abundance of unlabeled data, this study developed the MCC-ST framework. The core logic of this framework involves leveraging the collective intelligence of ensemble learning to mitigate the inherent bias of any single model. By iteratively expanding a high-quality pseudo-labeled dataset, the framework enhances the overall classification accuracy. A set of base classifiers, each based on distinct mathematical principles, is selected. A pseudo-label is assigned to an unlabeled sample only when a consensus is reached among all base classifiers. Let the MCC-ST framework consist of *N* diverse base classifiers {*M_1_*, *M_2_*,…, *M_N_*}. For a specific unlabeled sample x with a true label y across *K* total classes, let *a_i_* represent the prediction accuracy of the ith base classifier *M_i_*. To simplify the mathematical derivation and explain the theoretical behavior of the consensus mechanism, the following idealized assumptions are formulated. It should be noted that these assumptions are used for probabilistic analysis rather than as strict prerequisites for the practical application of MCC-ST.

Assumption A: Under the idealized analytical condition, the predictions and prediction errors of the base classifiers are assumed to be mutually independent.

Assumption B: When a model predicts incorrectly, the probability of misclassifying the sample into the remaining *K-1* incorrect categories is equal.

According to the framework logic, the condition for sample *x* to be assigned the pseudo-label *y* is that the prediction results of all models are consistent. The following probabilities are obtained:

##### 2.4.3.1 Probability of assigning the correct pseudo-label (*P_c_*)

The formula for calculating the probability that all models reach a consensus and the prediction result is equal to the true label y is shown in Equation 2.

(2) $$P_{c}=\prod_{i=1}^{N}a_{i}$$

##### 2.4.3.2 Probability of assigning an incorrect pseudo-label (*P_e_*)

All models reach a consensus, but the prediction result is an incorrect label *y*, ( ${\text{y}}^{,}\neq \text{y}$). For any specific incorrect category *k* (where k≠y), the formula for the probability that model *M_i_* predicts this category is shown in Equation 3.

(3) $$\begin{array}{c} P=\frac{1-a_{i}}{K-1} \end{array}$$

The formula for the probability that all models simultaneously predict a specific incorrect category *k* is shown in Equation 4.

(4) $$\begin{array}{c} P=\prod_{i=1}^{N}(\frac{1-a_{i}}{K-1}) \end{array}$$

Since there are *K*-*1* possible incorrect categories, the formula for calculating the total probability of consistent incorrect predictions is shown in Equation 5.

(5) $$\begin{array}{c} P_{e}=(K-1)\times \prod_{i=1}^{N}(\frac{1-a_{i}}{K-1})=\frac{\prod_{i=1}^{N}(1-a_{i})}{{\left(K-1\right)}^{N-1}} \end{array}$$

##### 2.4.3.3 Probability of not assigning a pseudo-label (*P_d_*)

According to the normalization property of probability, a sample is either assigned a correct label, assigned an incorrect label, or returned to the unlabeled pool due to inconsistent model results. Therefore, the formula for calculating the probability of not assigning a pseudo-label is shown in Equation 6.

(6) $$P_{d}=1-P_{c}-P_{d}=1-[\prod_{i=1}^{N}(\frac{1-a_{i}}{K-1})+\frac{\prod_{i=1}^{N}(1-a_{i})}{{\left(K-1\right)}^{N-1}}]$$

##### 2.4.3.4 Signal-to-noise ratio of pseudo-labels

Based on the defined formulas for *P_c_* and *P_e_*, let the signal-to-noise ratio of the pseudo-labels be *R*; the formula for calculating *R* is shown in Equation 7.

(7) $$\begin{array}{c} R=\frac{P_{c}}{P_{e}}=\frac{\prod_{i=1}^{N}a_{i}}{\frac{\prod_{i=1}^{N}(1-a_{i})}{{\left(K-1\right)}^{N-1}}}={\left(K-1\right)}^{N-1}\times \prod_{i=1}^{N}(\frac{a_{i}}{1-a_{i}}) \end{array}$$

Based on the aforementioned formulas, the influence of three key parameters on the signal-to-noise ratio of pseudo-labels was analyzed. For ease of analysis, it is assumed that the minimum accuracy of all base models is a and that all models operate at this minimum standard ( $a_{i}=a$). Under these conditions, the formula can be simplified as shown in Equation 8.

(8) $$R={\left(K-1\right)}^{N-1}\times {\left(\frac{a}{\left(1-a\right)}\right)}^{N}=\frac{1}{\left(K-1\right)}\times {\left[(K-1)\times \frac{a}{\left(1-a\right)}\right]}^{N}$$

Since K is a fixed value, when $\frac{\text{a}}{\left(1-\text{a}\right)}>1\;(\text{a}>0.5$), increasing the number of models *N* will cause the ratio *R* to increase exponentially. This means that increasing the number of models can significantly filter out noise, which is the model consensus mechanism proposed in this study. Meanwhile, it should be noted that when there are too many base models, *P_d_* will approach 1, resulting in the framework being unable to generate pseudo-labels. Therefore, it is necessary to select an appropriate number of models based on model accuracy.

Assuming the initial accuracy of the model is 0.6, with 6 categories and 3 base models, R=84.375. This indicates that the pseudo-labels assigned to the unlabeled data are highly likely to be correct. However, $P_{d}\approx 0.8$, there is an 80% probability that labels will not be assigned. If all unlabeled data are input into the framework simultaneously, only a small fraction may generate pseudo-labels, while the rest remains unlabeled. Consequently, this study introduces a batch cyclic self-training mechanism. In each iteration, a batch of data is selected from the unlabeled dataset for processing. The data assigned with pseudo-labels are combined with the data used to train the base models in the previous iteration to retrain the base models, followed by the generation of pseudo-labels for the next set of data. Due to the expansion of the training set and its high data quality, the model accuracy is theoretically expected to improve. Assuming that the model precision exhibits logarithmic growth to simulate a trend where the improvement rate gradually slows down, let the initial accuracy be *a* and the growth coefficient be *b*. The formula for calculating the accuracy of the base models in the *t-th* round is shown in Equation 9.

(9) $$\begin{array}{c} a^{\left(t\right)}=a+(b\times ln(1+t)) \end{array}$$

The formula for calculating the probability of the framework correctly assigning pseudo-labels is shown in Equation 10.

(10) $$\begin{array}{c} P_{c}^{\left(t\right)}={\left(a+(b\times ln(1+t))\right)}^{N} \end{array}$$

The formula for calculating the probability that the framework incorrectly assigns pseudo-labels is shown in Equation 11.

(11) $$\begin{array}{c} P_{e}^{\left(t\right)}=\frac{{\left(1-(a+(b\times ln(1+t)))\right)}^{N}}{{\left(K-1\right)}^{N-1}} \end{array}$$

At this point, the probability of the data being returned in the *t-th* round is calculated as shown in Equation 12.

(12) $$\begin{array}{c} P_{d}^{\left(t\right)}=1-[(a+(b\times ln(1+t)){)}^{N}+\frac{{\left(1-(a+(b\times ln(1+t)))\right)}^{N}}{{\left(K-1\right)}^{N-1}}] \end{array}$$

**Figure 4:**
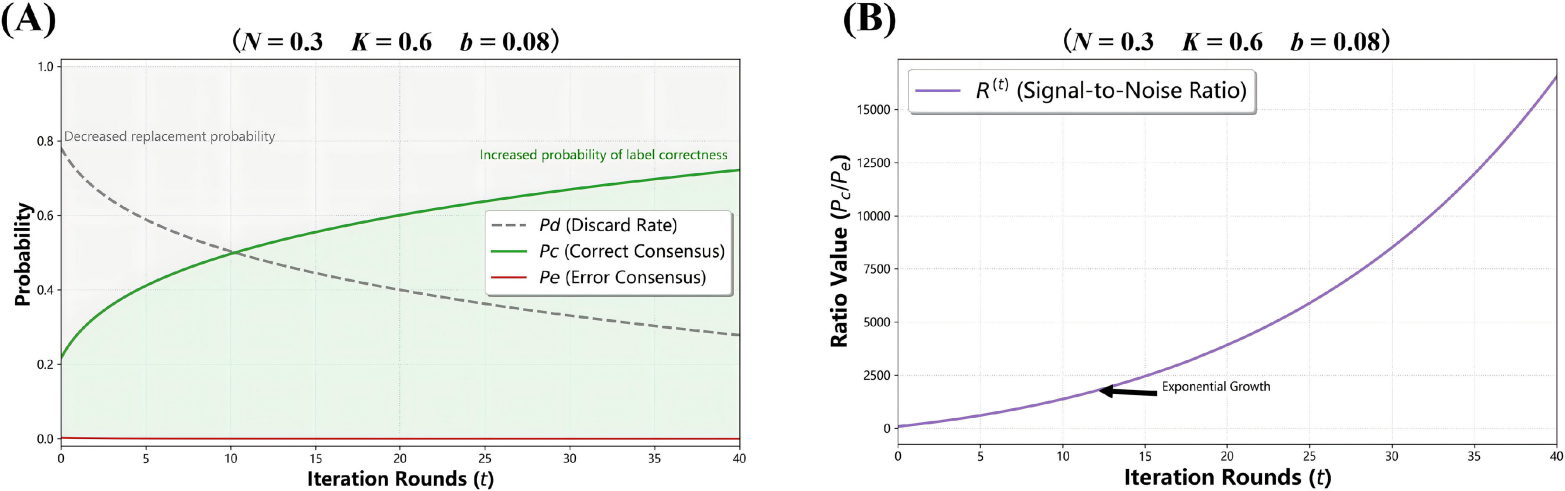
Theoretical changes in pseudo-labeling probabilities during MCC-ST iteration. **(A)** Changes in the probability of correct pseudo-labeling, incorrect pseudo-labeling, and non-labeling during cyclic self-training. **(B)** Change in the signal-to-noise ratio of pseudo-labels during iteration under the idealized analytical assumptions.

When a=0.6, N=3 and  K=6, the function plots of $P_{c}^{\left(t\right)}$, $P_{e}^{\left(t\right)}$, $P_{d}^{\left(t\right)}$ and *R* are shown in **Figure 4**.

**Figure 5:**
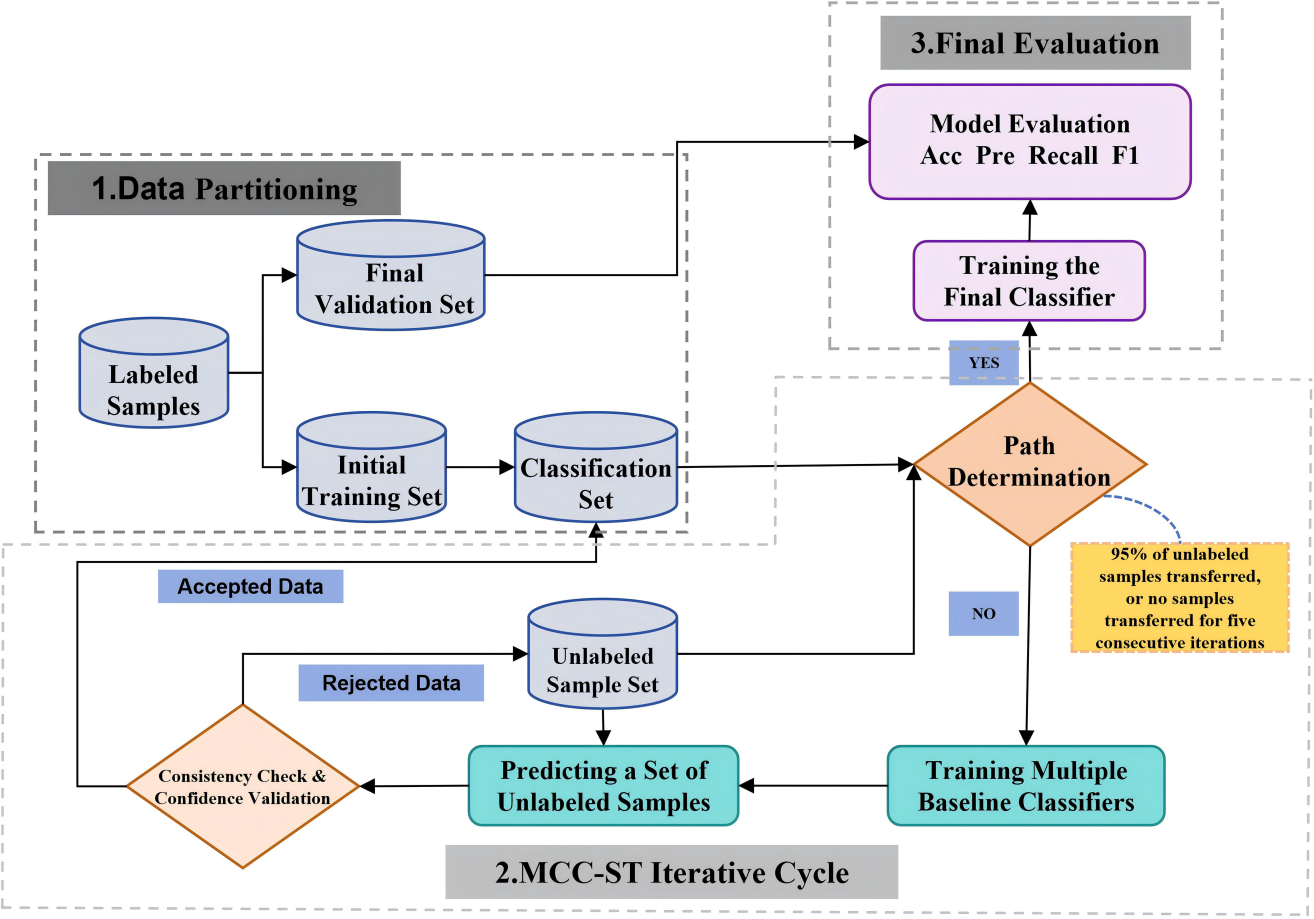
Schematic diagram of the multi-model collaborative cyclic self-training framework.

The results demonstrate that $P_{c}^{\left(t\right)}$, $P_{e}^{\left(t\right)}$, $P_{d}^{\left(t\right)}$ and *R* all exhibit monotonicity. Throughout the iterative cyclic training process, the probability of the framework assigning labels to the data progressively increases, characterized by a significant rise in accuracy and a decline in the error rate. Moreover, the signal-to-noise ratio increases quasi-exponentially as the number of iterations grows. The iteration process terminates when 95% of the unlabeled data has been converted into pseudo-labeled data or when no pseudo-labels are generated for five consecutive cycles. Using the final augmented dataset, a classifier with an architecture entirely distinct from the base models used within the framework is trained to serve as the final disease grading model. Its performance is then evaluated on an independent validation set. The specific structural flowchart of the MCC-ST framework is shown in **Figure 5**.

The implementation logic of MCC-ST was further specified as follows. The framework consists of multiple heterogeneous base classifiers for pseudo-label generation and one or more final classifiers for RCDI-based severity grading. In each iteration, a fixed batch of severity-unlabeled samples was selected from the unlabeled pool and predicted by all base classifiers. A pseudo-label was assigned only when all base classifiers predicted the same RCDI severity class; otherwise, the sample was returned to the unlabeled pool. No held-out validation samples were used during pseudo-label generation or pseudo-label selection. After each iteration, newly accepted pseudo-labeled samples were added to the training set, and the base classifiers were retrained before the next iteration. The iteration stopped when 95% of the severity-unlabeled samples had been converted into pseudo-labeled samples or when no pseudo-labels were generated for five consecutive iterations.

#### 2.4.4 Model implementation, validation strategy, and evaluation metrics

All machine-learning analyses were implemented in Python 3.9 using scikit-learn 1.7.2, NumPy 2.4.6, pandas 3.0.3, and related scientific computing packages. The candidate classifiers included K-nearest neighbors (KNN), decision tree (DT), random forest (RF), support vector machine (SVM), and logistic regression (LR). To improve reproducibility, all random processes, including fold generation and model initialization where applicable, were controlled by fixed random seeds.

Before model training, the selected vegetation indices were standardized using z-score normalization. For each cross-validation split, the scaler was fitted only on the training fold and then applied to the corresponding validation fold to avoid information leakage. Feature standardization was applied to all models to maintain a consistent preprocessing procedure.

Hyperparameter tuning was first performed using grid search to determine the optimal parameter combination for each model. The optimal hyperparameters obtained from grid search were then fixed and used in the subsequent repeated cross-validation evaluation. The repeated cross-validation stage was designed to evaluate the stability and robustness of the selected model settings under different random data partitions, rather than to further optimize hyperparameters.

For the binary classification, model performance was evaluated using five-fold cross-validation. In each repetition, the dataset was divided into five folds while preserving the class distribution in each fold as much as possible. Four folds were used for model training and the remaining fold was used for validation. This five-fold process was repeated 30 times using different random seeds.

For the MCC-ST semi-supervised severity grading framework, model performance was evaluated using three-fold cross-validation. This strategy was adopted because only 73 RCDI-labeled samples were available for six severity classes, and five-fold splitting could lead to extremely small validation subsets or insufficient class representation in some folds. In each repetition, the labeled dataset was divided into three stratified folds. Two folds of the labeled samples were used for MCC-ST training and pseudo-label generation, while the remaining labeled fold was retained as the validation set. The validation fold was not involved in pseudo-label generation or model training.

To further evaluate the stability of the classification results, repeated stratified K-fold cross-validation was conducted 30 times. In each repetition, the dataset was randomly repartitioned into K folds while preserving the class distribution, and accuracy, precision, recall, and F1-score were reported as mean ± standard deviation. In addition, a permutation test was performed by randomly shuffling the binary labels while keeping the vegetation-index features unchanged. The same classifiers and validation procedure were repeated 30 times, and the permuted-label performance was used as a baseline for assessing whether the true-label classification results were driven by non-random spectral-label associations.

In this study, accuracy, precision, recall, and F1-score are employed for binary classification tasks. For multi-class classification, weighted averages of these metrics are used, alongside confusion matrices, to focus on the analysis of misclassifications between different grades.

Accuracy is one of the metrics for measuring model performance, which is the ratio of the number of correctly identified samples to the total number of samples. Generally, the higher the accuracy of the model, the better the performance; the calculation formula is shown in Equation 13.

(13) $$\begin{array}{c} Accuracy=\frac{TP+TN}{TP+FN+FP+TN} \end{array}$$

Where TP represents True Positives, the number of samples correctly predicted as positive by the model. FP represents False Positives, the number of negative samples incorrectly predicted as positive by the model. FN represents False Negatives, the number of positive samples incorrectly predicted as negative by the model. TN represents True Negatives, the number of samples correctly predicted as negative.

Precision is the proportion of samples predicted as positive by the model that are actually positive. The calculation formula is shown in Equation 14.

(14) $$\begin{array}{c} Precision=\frac{TP}{TP+FP} \end{array}$$

Recall represents the proportion of samples predicted as positive by the model out of all actual positive samples. The calculation formula is shown in Equation 15.

(15) $$\begin{array}{c} Recall=\frac{TP}{TP+FN} \end{array}$$

F_1_-score is a comprehensive metric that balances the influence of precision and recall, providing a more comprehensive evaluation of classifier performance. A higher F_1_-score indicates superior model quality. The calculation formula is shown in Equation 16.

(16) $$\begin{array}{c} F_{1}=\frac{2*precision*recall}{precision+recall} \end{array}$$

The MCC-ST models were evaluated using 30 random-seed repeated stratified three-fold cross-validations based on the 73 ground RCDI sampling points. In each fold of the MCC-ST evaluation, the held-out ground RCDI samples were used only for validation and were not involved in model training, pseudo-label generation, or pseudo-label selection. Pseudo-labeling was performed only using the training portion of the ground RCDI samples and the severity-unlabeled random samples. Because the cross-validation was performed at the sample level rather than using plot-level or date-level grouped splitting, samples from the same plot or sampling date could appear in both the training and validation folds. The performance of the grading models was summarized as mean ± standard deviation, and the 95% confidence intervals of accuracy were estimated from the repeated validation results to quantify the statistical uncertainty caused by the small labeled dataset. These repeated-validation results were used to assess within-dataset stability rather than external generalization.

## 3 Results

### 3.1 Screening results of sensitive vegetation indices and physiological response analysis

#### 3.1.1 Analysis of probability density overlap and point-biserial correlation

**Figure 6:**
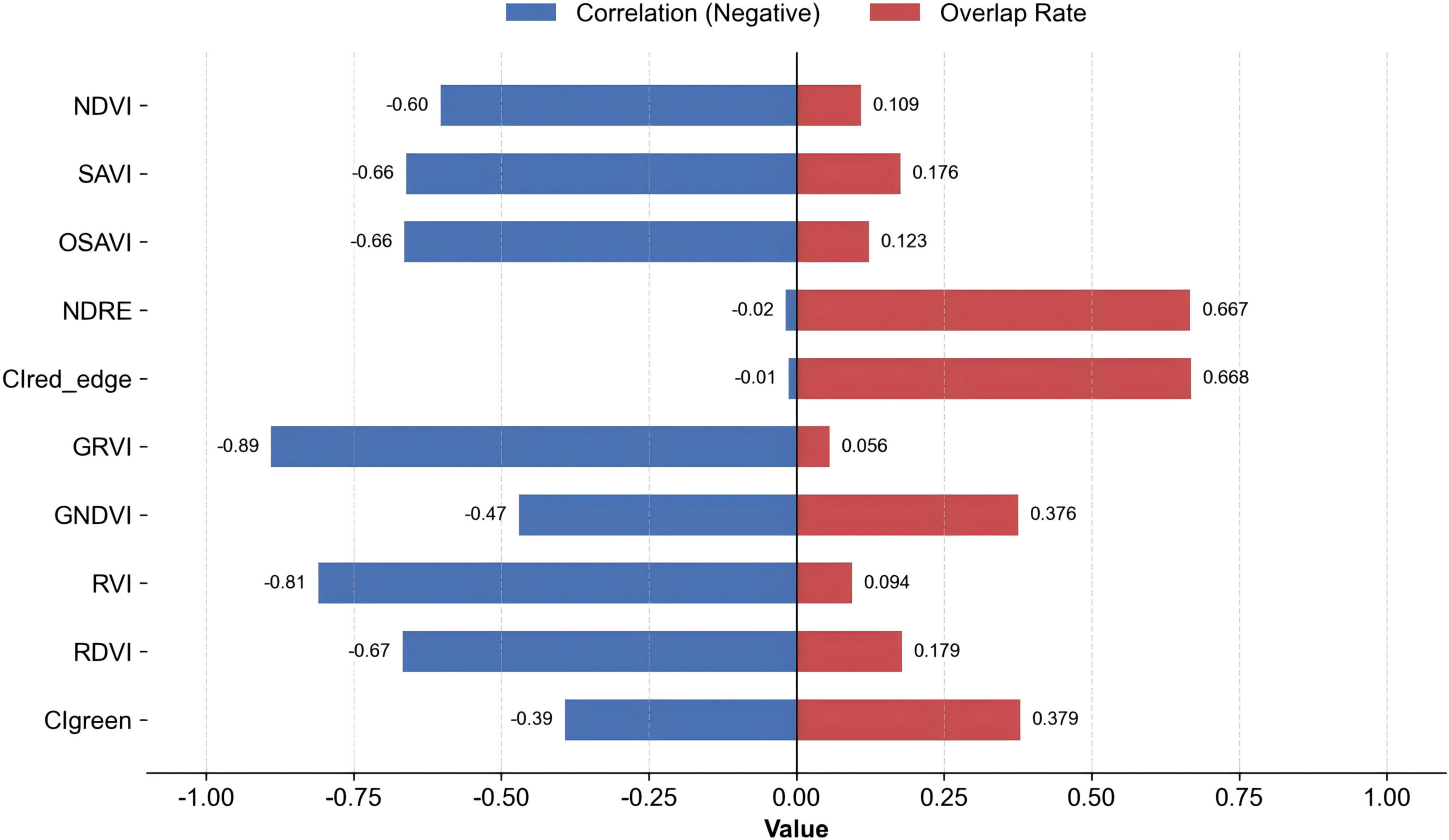
Feature separability and point-biserial correlation analysis of candidate vegetation indices. The overlap rate based on kernel density estimation indicates the distributional separability between normal and BER-symptomatic samples, while the point-biserial correlation quantifies the association between each vegetation index and the binary BER label.

Objects of different categories, such as normal and BER-symptomatic plants, often exhibit distinct probability distributions in specific spectral indices. Through the kernel density estimation method, this study smooths discrete sampling point data into continuous density curves to calculate the overlap rate between the curves. The point-biserial correlation coefficient provides a rigorous quantitative verification of the linear dependency between candidate features and binary disease labels, confirming which features can serve as predictors for classification and ensuring that subsequent models prioritize core variables with the strongest capability to capture early weak signals. The specific results are shown in **Figure 6**.

Among the 10 selected vegetation indices, NDVI, SAVI, OSAVI, GRVI, RVI, and RDVI exhibit significant inter-class separation characteristics, indicating that the numerical distributions of the two sample categories are widely dispersed along the axis. These indices can sensitively capture physiological changes caused by diseases and possess strong discriminative power. In contrast, NDRE, CIred-edge, CIgreen, and GNDVI show limitations of excessively high overlap in the PDF analysis, indicating that the values of the two sample categories on these indices are very close and the spectral features are confounded. This means these indices are insensitive to diseases and cannot effectively distinguish between the two. Vegetation indices demonstrating significant inter-class separation are all significantly linearly correlated with the binary disease labels, whereas indices with obviously high overlap rates show very poor correlation, with the two methods yielding highly consistent conclusions. Specifically, NDVI, SAVI, OSAVI, GRVI, RVI, and RDVI showed the lowest inter-class overlap in the feature space and strong correlations with disease labels. The sensitive feature set selected through the dual-criteria screening eliminates redundancy and noise from the data, providing a high-quality data foundation and interpretability guarantee for the subsequent construction of high-precision BER monitoring models.

#### 3.1.2 Correlation analysis among vegetation indices

**Figure 7:**
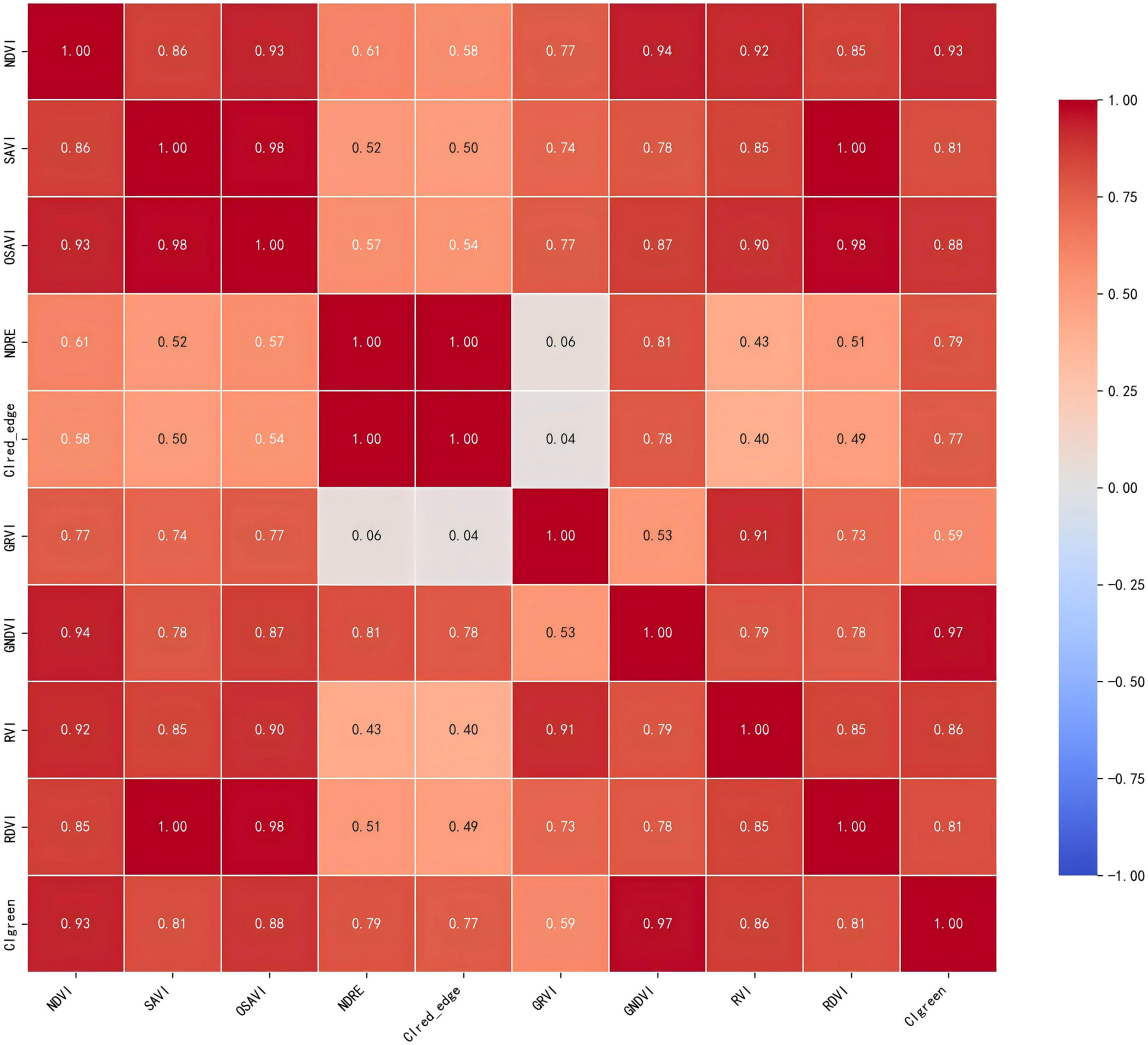
Pearson correlation analysis among candidate vegetation indices. The correlation matrix was used to evaluate redundancy among sensitive vegetation indices and to support the final selection of GRVI, NDVI, and SAVI as the optimized feature set.

While the six indices—NDVI, SAVI, OSAVI, GRVI, RVI, and RDVI—were selected in the aforementioned research, these multiple vegetation indices are essentially different mathematical combinations of the same set of spectral bands, resulting in extremely high correlations among them. To mitigate the interference of multicollinearity on the machine learning models, this study further constructed a Pearson correlation coefficient matrix to conduct an in-depth analysis of the redundancy among these highly sensitive features. The specific results of the correlation analysis are shown in **Figure 7**.

Analysis revealed that although many indices performed excellently in Section 3.1.1, they often exhibited extremely high correlations, indicating that their feature information is highly redundant. After redundant indices were removed using the threshold of r > 0.9, GRVI, NDVI, and SAVI showed relatively low inter-correlations. These three indices provided complementary information and were therefore retained as the final feature combination. Ultimately, GRVI, NDVI, and SAVI were retained as the final feature combination. This selection was not based solely on the numerical correlation threshold, but also on the complementary physiological and spectral meanings of the indices. GRVI is sensitive to green-red color variation and can capture canopy color changes associated with chlorosis or necrosis. NDVI reflects canopy vigor, vegetation coverage, and biomass-related changes caused by leaf rolling or canopy deformation. SAVI introduces soil-background adjustment and is particularly useful in processing tomato fields where soil, plastic film, and canopy gaps may influence early-stage UAV spectral signals. Therefore, the retained feature set balances disease sensitivity, redundancy control, physiological interpretability, and robustness to background interference.

### 3.2 Analysis of BER occurrence classification and severity grading models based on optimized vegetation indices

#### 3.2.1 Construction and evaluation of BER occurrence classification models

Using the three optimized vegetation indices, 550 binary-labeled data points were input into machine-learning models for binary classification evaluation through 30 repeated stratified five-fold cross-validations, and the results are shown in **Table 5**. As shown in **Table 5**, all five classifiers maintained stable binary classification performance across 30 repeated stratified five-fold cross-validations. The mean accuracies ranged from 96.91% to 97.64%, and the standard deviations were low, ranging from 0.13% to 0.54%. Among the tested models, RF achieved the highest mean accuracy of 97.64% ± 0.13% and the highest F1-score of 0.9847 ± 0.0008. These results indicate that the high binary classification performance was not caused by a single favorable data split, but reflected stable within-dataset separability between healthy and BER-stressed samples.

**Table 5:** Binary classification performance under 30 repeated five-fold cross-validations and permutation testing.

Model	ACC mean ± SD	PRE mean ± SD	Recall mean ± SD	F1 mean ± SD	Permuted ACC
K-Nearest Neighbors (KNN)	97.09% ± 0.37%	98.35% ± 0.33%	97.88% ± 0.34%	0.9811 ± 0.0024	72.59%
Decision Tree (DT)	97.45% ± 0.30%	98.58% ± 0.19%	98.12% ± 0.34%	0.9835 ± 0.0020	63.85%
Random Forest (RF)	97.64% ± 0.13%	98.59% ± 0.16%	98.35% ± 0.16%	0.9847 ± 0.0008	70.40%
Support Vector Machine (SVM)	96.91% ± 0.53%	98.11% ± 0.46%	97.88% ± 0.46%	0.9800 ± 0.0034	77.27%
Logistic Regression (LR)	96.91% ± 0.54%	97.89% ± 0.66%	98.12% ± 0.20%	0.9800 ± 0.0035	77.27%

The permutation test further showed that the permuted-label accuracies ranged from 63.85% to 77.27%, which were substantially lower than the true-label accuracies. The accuracy gaps between true-label and permuted-label models ranged from 19.64 to 33.60 percentage points. Notably, the highest permuted-label accuracy was 77.27% for SVM and LR. Considering the imbalanced binary label distribution shown in **Figure 3A**, this value mainly reflects the majority-class baseline rather than meaningful disease discrimination. Therefore, the high true-label performance was mainly supported by discriminative spectral information in GRVI, NDVI, and SAVI rather than by random dataset-specific label-feature associations.

Meanwhile, according to the mean feature importance analysis, GRVI (0.4213) > NDVI (0.4046) > SAVI (0.1741), indicating that GRVI and NDVI were the core contributing factors for classification. This suggests that the combined spectral features of GRVI, NDVI, and SAVI provide complementary information for distinguishing BER-stressed plants from healthy plants within the current dataset.

#### 3.2.2 Comparative evaluation of disease grading models based on RCDI and conventional DI

Using the three optimized vegetation indices, 73 data samples with different disease grade labels were input into machine learning models for multi-classification evaluation through three-fold cross-validation, and the results are shown in **Table 6**.

**Table 6:** Evaluation results for the grading of blossom-end rot in processing tomatoes.

Model	Mean accuracy	Mean precision	Mean recall	Mean F_1_-score
KNN	68.33%	72.73%	68.33%	66.77%
DT	64.39%	67.88%	64.39%	63.33%
RF	72.67%	72.18%	73.35%	72.67%
SVM	73.89%	76.53%	73.89%	72.88%
LR	72.50%	71.36%	72.50%	71.04%

The results indicate that using 73 graded data samples, some models are already capable of extracting features for different disease levels and performing a preliminary classification, which suggests that the selected vegetation index combination has potential for BER severity grading. However, the classification performance fails to meet practical requirements, primarily because the volume of training data is too small to accurately capture the variation trends of vegetation index combinations across different disease levels. This necessitates the acquisition of more data with disease grade labels to train the models. The results in **Table 6** also provided the quantitative basis for selecting base classifiers in the subsequent MCC-ST framework: models with acceptable initial grading ability and different learning mechanisms were preferred to ensure both pseudo-labeling reliability and classifier diversity. Although the sample distribution among BER severity levels was explicitly shown in **Figure 3B**, the relatively small number of samples in some classes, especially level_5, may still affect the stability of severity grading and the recognition performance of underrepresented classes.

To directly examine whether RCDI provides additional value over the conventional fruit-incidence-based DI, the same 73 ground sampling points were further labeled using DI and evaluated with the same machine-learning classifiers. As shown in **Table 7**, when conventional DI was used as the disease grading label, the best-performing model among the five classifiers was LR, with an accuracy of 0.5640, precision of 0.5457, recall of 0.5640, and F1-score of 0.5239. The remaining models showed even lower grading performance, with accuracies ranging from 0.4171 to 0.5329. Compared with the RCDI-based grading results, the DI-based models showed weaker compatibility with UAV-derived vegetation indices. This indicates that fruit-incidence-only DI is insufficient to fully represent the canopy-level spectral response of BER, whereas RCDI provides a more remote-sensing-compatible disease severity label by incorporating leaf rolling and young-leaf color information.

**Table 7:** Cross-validation performance of disease grading models using conventional DI as the target label.

Model	Mean accuracy	Mean precision	Mean recall	Mean F_1_-score
KNN	51.63%	48.80%	51.63%	0.4773
DT	41.71%	42.74%	41.71%	0.3938
RF	51.91%	52.00%	51.91%	0.4955
SVM	53.29%	49.13%	53.29%	0.489
LR	56.40%	54.57%	56.40%	0.5239

#### 3.2.3 Analysis of results for the MCC-ST semi-supervised classification framework

Due to the limited number of ground-truth labeled samples (N = 73), a stratified three-fold cross-validation strategy was adopted to avoid random bias resulting from a single data partition and to comprehensively evaluate the generalization stability of the MCC-ST framework under different data distributions. Due to the limited number of ground-truth RCDI severity-labeled samples (N = 73), the MCC-ST framework was evaluated using two complementary validation strategies. First, an optimized grid-search parameter-tuning strategy was used to compare the supervised baselines, conventional semi-supervised learning methods, and the proposed MCC-ST framework under the selected parameter configurations. Second, 30 random-seed repeated stratified three-fold cross-validations were conducted to quantify model variability and statistical uncertainty under different random data partitions. During the iterative process, batches of 20 unlabeled data points were processed per cycle, with GRVI, NDVI, and SAVI consistently utilized as features throughout the model training. Based on the initial BER severity grading results in **Table 6**, LR, SVM, and KNN were selected as the base classifiers of the MCC-ST framework for two reasons. First, these three models showed acceptable initial grading performance under the limited labeled samples, with accuracies of 72.50%, 73.89%, and 68.33%, respectively, indicating that each model had a basic ability to generate informative pseudo-labels. Second, they represent different learning mechanisms: LR is a probabilistic linear classifier, SVM is a maximum-margin classifier, and KNN is a distance-based non-parametric classifier. This model heterogeneity was expected to increase prediction diversity and reduce the risk of single-model confirmation bias. In contrast, DT and RF were not used as base classifiers because of their similar tree-based decision mechanisms; instead, they were retained as final classifiers to independently evaluate the pseudo-labeled dataset generated by MCC-ST.

To comprehensively evaluate the performance advantages of the MCC-ST framework under small-sample conditions and verify the necessity of the multi-model consensus mechanism in suppressing pseudo-label noise, this study designed comparative experiments including supervised learning baselines and mainstream semi-supervised learning methods. The experiments were conducted under identical stratified three-fold cross-validation data partitions, using 2/3 of the ground-truth labeled data and all unlabeled data for training, while the remaining 1/3 of the labeled data was used for validation. The experiments were conducted under identical stratified three-fold cross-validation partitions of the 73 ground RCDI sampling points. In each fold, approximately two-thirds of the ground RCDI samples were used as the severity-labeled training set, while the remaining one-third was strictly held out for validation. The held-out validation samples were not used during pseudo-label generation, pseudo-label filtering, model retraining, or final model training. The 550 random sampling points were treated as severity-unlabeled samples for pseudo-label generation within each training fold.

(1) Baseline Supervised Learning (Baseline): Only the original small set of labeled samples (N = 73) was used to directly train the final classifiers (DT and RF), serving as the lower-bound reference for performance evaluation.

(2) Standard Self-Training: Individual KNN, SVM, and LR models were employed as teacher models. Each model generated pseudo-labels based on its own high-confidence predictions to augment the training set.

(3) Standard Co-Training: A classic dual-view collaborative strategy was adopted, constructing model combinations of KNN+SVM, KNN+LR, and SVM+LR. The two models exchanged high-confidence samples for training.

(4) MCC-ST Framework (Ours): The triple-model consensus mechanism (KNN+SVM+LR) proposed in this study was employed to execute rigorous pseudo-label filtering and cyclic iteration.

**Figure 8:**
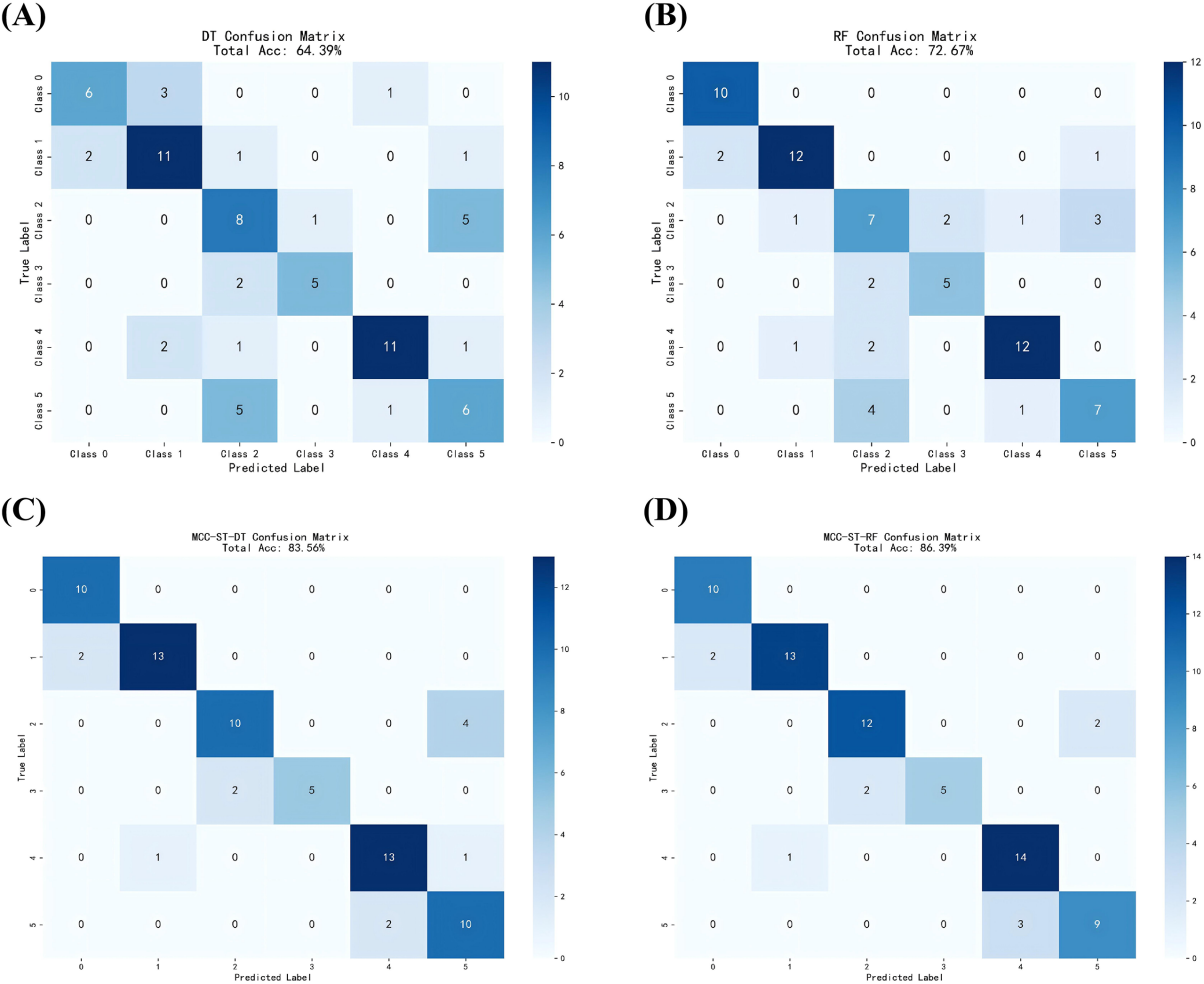
Confusion matrices of BER severity-grading models. **(A)** DT; **(B)** RF; **(C)** MCC-ST + DT; and **(D)** MCC-ST + RF. The matrices compare the classification results of the baseline models and models trained with the MCC-ST-augmented dataset, showing the distribution of correct and incorrect predictions across different BER severity levels.

All experiments utilized DT and RF as the final RCDI-based disease grading models, and adopted Accuracy, Precision, Recall, and F1-score as evaluation metrics. The optimized grid-search parameter-tuning comparison results are shown in **Table 8**. The confusion matrices of the initially trained classification models and the classification models trained using the final framework are shown in **Figure 8**.

**Table 8:** Evaluation results of different models based on optimized parameters.

Method	Base classifiers	Final model	Accuracy	Precision	Recall	F_1_-score
Baseline (Supervised)	None	DT	64.39%	67.88%	64.39%	63.33%
Standard Self-Training	KNN	DT	60.33%	57.71%	60.33%	57.61%
Standard Self-Training	SVM	DT	62.89%	68.03%	62.89%	61.03%
Standard Self-Training	LR	DT	66.89%	69.03%	66.89%	67.94%
Standard Co-Training	KNN+SVM	DT	69.83%	73.28%	69.83%	68.99%
Standard Co-Training	KNN+LR	DT	68.44%	70.31%	68.44%	68.11%
Standard Co-Training	SVM+LR	DT	69.94%	72.65%	69.94%	68.04%
MCC-ST (Ours)	KNN+SVM+LR	DT	83.56%	85.87%	83.56%	83.37%
Baseline (Supervised)	None	RF	72.67%	72.18%	73.35%	72.67%
Standard Self-Training	KNN	RF	69.89%	67.76%	69.89%	66.52%
Standard Self-Training	SVM	RF	72.67%	74.16%	72.67%	72.29%
Standard Self-Training	LR	RF	74.00%	75.23%	74.00%	73.47%
Standard Co-Training	KNN+SVM	RF	74.06%	75.61%	74.06%	72.46%
Standard Co-Training	KNN+LR	RF	73.94%	76.94%	73.94%	73.17%
Standard Co-Training	SVM+LR	RF	74.11%	74.27%	74.11%	73.27%
MCC-ST (Ours)	KNN+SVM+LR	RF	86.39%	87.96%	86.39%	86.05%

In the optimized grid-search parameter-tuning comparison shown in **Table 8**, results indicate that whether using DT or RF as the final classifier, MCC-ST achieved the best performance metrics overall, significantly outperforming baseline supervised learning and traditional semi-supervised learning methods. On the DT model, the accuracy of MCC-ST reached 83.56%, an increase of 19.17% compared to the Baseline; on the RF model, the accuracy of MCC-ST reached 86.39%, an increase of 13.72% compared to the Baseline. This strongly demonstrates that the multi-model consensus mechanism introduced by MCC-ST can effectively filter noise in pseudo-labels, and the quality of the generated training data far exceeds that of traditional methods.

When performing Self-Training on models individually, the accuracies on both DT and RF were even lower than the Baseline. This confirms that single-model self-training is prone to confirmation bias. If the initial model, trained with only 73 samples, makes incorrect predictions and incorporates these erroneous pseudo-labels into the training set, the error will be amplified during iterations, leading to a decline rather than an improvement in model performance. This underscores the necessity of the multi-model collaborative noise filtering employed by MCC-ST. Although Co-Training significantly improved accuracy compared to the Baseline, the magnitude of improvement was not as large as that of MCC-ST. This indicates that while consensus mechanisms can improve the quality of pseudo-labels, the view difference between models may be insufficient when feature dimensionality is low and the number of models is limited, leading them to make mistakes simultaneously and failing to form effective complementarity, thereby restricting further improvement in pseudo-label quality.

To further evaluate whether the performance improvement of MCC-ST was stable under different random data partitions, 30 random-seed repeated stratified three-fold cross-validations were conducted for the original DT/RF models and the MCC-ST-enhanced DT/RF models. As shown in **Table 9**, the original DT and RF models achieved accuracies of 60.91% ± 3.94% and 68.88% ± 4.00%, respectively. After MCC-ST-based pseudo-label augmentation, MCC-ST + DT and MCC-ST + RF achieved accuracies of 85.00% ± 0.31% and 85.07% ± 0.42%, respectively. Compared with the corresponding original models, the repeated-validation accuracy increased by 24.09 percentage points for DT and 16.19 percentage points for RF. These results indicate that the performance improvement of MCC-ST was not dependent on a single optimized split, but remained consistent under repeated random-seed validation.

**Table 9:** Performance uncertainty of DT/RF grading models under 30 random-seed repeated stratified three-fold cross-validations.

Model	Accuracy mean ± SD	95% CI of accuracy	Precision mean ± SD	Recall mean ± SD	F1-score mean ± SD
Original DT	60.91% ± 3.94%	59.50%–62.32%	62.11% ± 4.04%	60.91% ± 3.94%	60.81% ± 4.03%
Original RF	68.88% ± 4.00%	67.45%–70.32%	69.42% ± 4.27%	68.88% ± 4.00%	68.52% ± 4.11%
MCC-ST + DT	85.00% ± 0.31%	84.89%–85.11%	85.38% ± 0.41%	85.00% ± 0.31%	84.84% ± 0.32%
MCC-ST + RF	85.07% ± 0.42%	84.92%–85.22%	85.39% ± 0.58%	85.07% ± 0.42%	84.88% ± 0.44%

**Figure 9:**
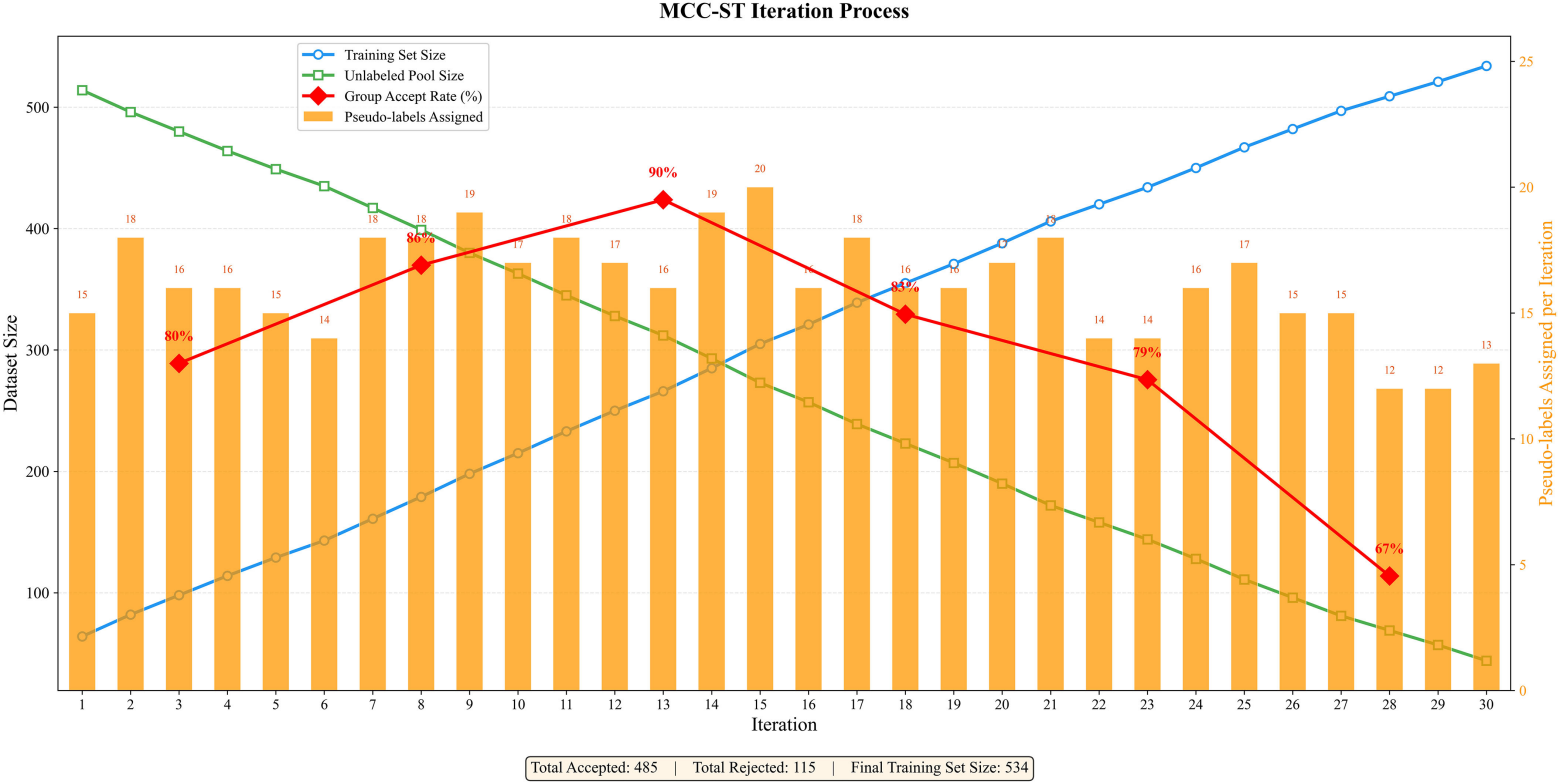
Representative iterative self-training process of the proposed MCC-ST framework.

To further clarify the pseudo-labeling process, the class distribution of pseudo-labeled samples generated by MCC-ST was recorded. Under the optimized parameter setting, MCC-ST generated more than 500 pseudo-labeled samples in total. As shown in **Table 10**, the generated pseudo-labeled samples covered all six RCDI severity classes and were mainly distributed across Classes 0–4, whereas Class 5 contained fewer pseudo-labeled samples. This distribution was consistent with the relatively limited occurrence of extremely severe samples in the field survey. To further illustrate the iterative self-training process of the proposed MCC-ST framework, a representative example of the pseudo-label generation procedure is presented in **Figure 9**. In the 30 random-seed repeated stratified three-fold cross-validations, the pseudo-label counts for each class also remained within a certain range. These results indicate that the consensus-based pseudo-labeling process did not simply assign all unlabeled samples to a dominant class, but generated pseudo-labeled samples across different RCDI severity levels.

**Table 10:** Class distribution of pseudo-labeled samples generated by MCC-ST.

RCDI severity class	Pseudo-label count under optimized parameters	Pseudo-label count range under 30 repeated validations
Class 0	120	110–122
Class 1	102	96–116
Class 2	105	89–113
Class 3	103	87–125
Class 4	84	62–89
Class 5	20	18–28

## 4 Discussion

### 4.1 Effectiveness of feature selection and discussion of binary classification results

This study adopted a three-level screening strategy comprising probability density distribution, point-biserial correlation, and feature redundancy analysis. From the 10 candidate vegetation indices, GRVI, NDVI, and SAVI were selected as the core feature set, achieving stable within-dataset binary classification performance with mean accuracies of 96.91%–97.64% across 30 repeated five-fold cross-validations.

The internal cause of BER is primarily the insufficient upward supply of calcium (Karlsons et al., 2023), while the external cause is mainly water stress (Millones-Chanamé et al., 2019). Based on the feature importance analysis, GRVI and NDVI contribute almost equally to the model, while SAVI remains indispensable. The specific analysis is as follows:

First, this study utilized leaf chlorosis/necrosis (L_C_) at the apex as a critical feature for determining calcium deficiency. When plants lack calcium, chlorophyll content decreases (Rangnekar, 1975), and the margins of new leaves often exhibit chlorosis and necrosis, leading to changes in leaf color. Since GRVI is sensitive to leaf color variations (Motohka et al., 2010), it serves as a key feature for BER identification. Second, leaf rolling (L_R_) was identified as an essential indicator of water stress. The curling of canopy leaves from a flat, expanded state into a cylindrical shape is a significant manifestation of water deficit; this rolling reduces the effective canopy coverage. Given that NDVI is an index reflecting crop growth and biomass (Carlson and Ripley, 1997), it is also highly effective for BER determination. Finally, the role of SAVI is to provide robust correction by eliminating background noise (Chen et al., 2020). Processing tomatoes are typically cultivated using plastic film mulching or wide-row patterns; during the early stages of disease onset, plant wilting or leaf rolling exposes more of the soil or plastic mulch background.

The robustness analysis further reduced the possibility that the high binary classification accuracy was only caused by a favorable split or random label-feature association. The low standard deviations across 30 repeated cross-validations indicated stable performance under different data partitions, while the permutation test showed that the true-label accuracies exceeded the permuted-label baselines by 19.64–33.60 percentage points.

In summary, the feature set comprising GRVI, NDVI, and SAVI showed stable within-dataset separability for binary BER classification. Furthermore, based on the 73 ground RCDI sampling points, the machine-learning models demonstrated preliminary potential for disease grading, although the limited number of accurately labeled severity samples still constrained grading performance.

Although GRVI, NDVI, and SAVI achieved satisfactory performance in this study, the exclusive use of vegetation indices as model inputs should be acknowledged as a limitation. Texture features derived from UAV imagery can provide additional information on canopy structure, spatial heterogeneity, and local stress patterns, which may be useful for characterizing leaf rolling and uneven canopy changes associated with BER. However, this study was conducted under a label-scarce condition, especially with only 73 RCDI-labeled samples for severity grading. Introducing a large number of texture variables would increase feature dimensionality and the risk of overfitting. Therefore, this study used a compact and physiologically interpretable vegetation-index feature set to improve model stability and interpretability.

### 4.2 Metric discussion on the leaf-fruit collaborative RCDI-based BER severity index

BER in processing tomatoes is essentially a physiological disorder triggered by localized calcium deficiency; traditional diagnostic criteria rely on necrotic and collapsed symptoms in the distal tissues of the fruit (Sethi et al., 2024). However, the construction of traditional disease indices is based solely on the observation of fruit lesions, which leads to a lag in the monitoring window and makes it difficult to establish a direct physical connection between ground-level symptoms and canopy remote sensing signals.

To address this limitation, this study proposed the Leaf-Fruit Collaborative Disease Index (RCDI), based on the pathogenesis of BER and the physiological mechanisms of long-distance calcium transport and distribution under water stress. The innovation of RCDI lies in the introduction of functional leaf rolling (L_R_) and young leaf color (L_C_) as core characterizations, establishing a strong correlation between the physiological manifestations of canopy leaves and the health status of the fruit. The indicators selected in this study have been theoretically and practically verified by an extensive body of research.

**Figure 10:**
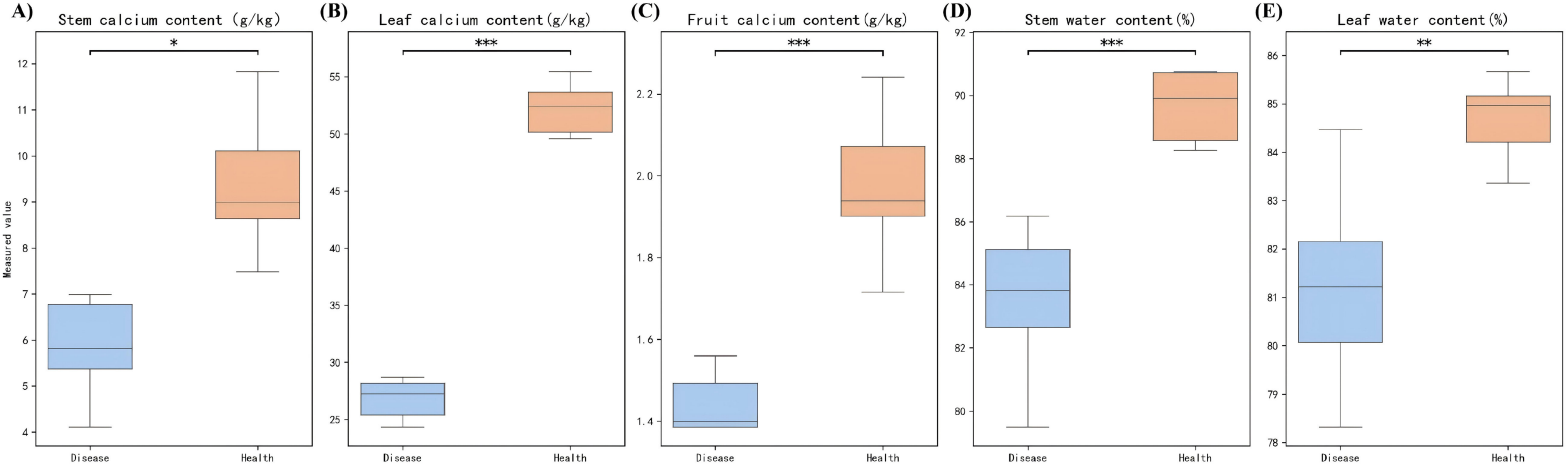
Physiological differences between normal and BER-symptomatic processing tomato plants. **(A–E)** show differences in stem total calcium content, leaf total calcium content, fruit total calcium content, stem water content, and leaf water content, respectively. These measurements were conducted on representative normal and BER-symptomatic plants.

To examine whether the canopy phenotypes incorporated into the RCDI were physiologically meaningful, water- and calcium-related traits were compared between healthy and BER-stressed processing tomato plants. As shown in **Figure 10**, BER-stressed plants showed significant changes in water and calcium-related physiological traits compared with healthy plants. Specifically, stem water content and leaf water content decreased significantly in BER-stressed plants, indicating that the stressed plants were affected by water deficit. This result provides physiological support for using functional leaf rolling (L_R_) as an indicator of water-stress-related canopy deformation. In addition, calcium-related traits also differed significantly between healthy and BER-stressed plants. The stem total calcium content, leaf total calcium content, and fruit total calcium content were all lower in BER-stressed plants than in healthy plants. In particular, the marked decrease in leaf total calcium content indicates that calcium deficiency was closely associated with the occurrence of BER in this study. Because calcium deficiency can induce chlorosis and necrosis in young leaves, this result supports the inclusion of emerging leaf color (L_C_) as a canopy-level indicator reflecting calcium-deficiency-related physiological stress. Overall, the physiological measurements shown in **Figure 10** provide supporting evidence that L_R_ and L_C_ are not arbitrary visual scores, but are related to the water and calcium physiological processes underlying BER.

In this study, BER-stressed plants showed significantly lower leaf water content than healthy plants, indicating that BER occurrence was accompanied by a decline in plant water status. One typical canopy manifestation of water deficit is leaf rolling or wilting. Previous research has established that leaf rolling serves to delay moisture loss (Yavas et al., 2023). Drought experiments have further confirmed that wilting and rolling in tomato leaves are highly correlated with water stress (Chiwina et al., 2024). Additionally, acoustic emission research on tomato plants indicates that drought leads to substantial xylem cavitation, which subsequently results in leaf wilting (Qiu et al., 2002). In summary, existing literature validates the strong correlation between water stress and leaf rolling in tomatoes. Given that water stress-induced xylem cavitation is a prominent trigger for BER, employing leaf rolling as an indicator for assessing BER severity is both scientifically sound and accurate.

Previous research has established that calcium deficiency is the primary factor in the occurrence of BER in tomato fruits (Rached et al., 2018). In this study, BER-stressed plants showed significantly lower leaf total calcium content than healthy plants, indicating that BER occurrence was accompanied by reduced calcium status in plant tissues. Research has demonstrated that significant necrosis can be observed on tomato leaves as early as the fifth day of Ca deficiency (Baboulène et al., 2007). Furthermore, subsequent analysis indicates that calcium deficiency leads to intumescence (swelling) injury on the surface of tomato leaves, which eventually results in leaf necrosis; notably, calcium levels are significantly correlated with the rate of leaf expansion injury (Sita, 2024). Consequently, calcium deficiency induces necrosis at the leaf margins or even across the entire leaf blade. Therefore, utilizing leaf color variation as an evaluation metric for BER severity effectively reflects the extent of calcium deficiency.

To further illustrate the temporal behavior of RCDI and conventional DI during BER development, three representative sampling points were selected for comparison between the early fruiting stage and the peak fruiting stage. The purpose of this comparison was not to provide an independent statistical validation, but to demonstrate how RCDI and DI respond differently under typical field situations, especially when fruit symptoms are absent, unstable, or not fully synchronized with canopy stress signals.

As shown in **Table 11**, P1 demonstrates the early-monitoring limitation of DI when no diseased fruits are observed: DI was 0 on June 20, whereas RCDI had already reached 0.24, and DI subsequently increased to 0.62 on July 16, suggesting that RCDI could capture canopy-level stress signals before visible fruit symptoms were fully expressed. P2 further shows the temporal instability of DI, as DI decreased from 0.57 to 0.50, which may be related to fruit developmental dynamics and the denominator effect of total fruit number rather than true disease alleviation; in contrast, RCDI increased from 0.34 to 0.55, showing a more consistent response to plant stress progression. Across the three cases, DI varied widely at the early fruiting stage, ranging from 0 to 0.57, whereas RCDI remained within a narrower range of 0.24–0.34 and increased to 0.55–0.61 at the peak fruiting stage. These representative cases suggest that RCDI is less affected by the occasional presence or absence of diseased fruits at a specific sampling time and can more stably characterize the transition from early canopy stress to later fruit-level BER manifestation. It should be noted that the RCDI severity thresholds proposed in this study are author-defined ordinal categories rather than officially established standards. They were designed to convert continuous RCDI values into reproducible severity labels for model training and field interpretation. Future multi-year and multi-site studies should further validate and refine these thresholds under different cultivars, cultivation systems, and environmental conditions.

**Table 11:** Temporal comparison of DI and RCDI at three representative sampling points.

Sampling point	June 20 DI	June 20 RCDI	July 16 DI	July 16 RCDI
P1	0.00	0.24	0.62	0.61
P2	0.57	0.34	0.50	0.55
P3	0.33	0.30	0.51	0.58

Compared to traditional evaluation systems that focus solely on fruit necrosis, RCDI integrates structural deformations induced by environmental triggers and symptomatic manifestations resulting from physiological stress. It possesses superior biological interpretability and timeliness, providing a solid theoretical foundation for the inversion of fruit-manifested abiotic physiological disorders using remote sensing techniques.

Because RCDI incorporates both fruit incidence and canopy symptoms, the prediction target in this study should be interpreted as RCDI-based BER severity rather than fruit-only BER incidence. This distinction is important because UAV multispectral imagery primarily captures canopy-level spectral responses and cannot directly observe fruit symptoms hidden beneath the canopy. Therefore, the purpose of RCDI is not to replace a fruit-only diagnostic endpoint, but to construct a remote-sensing-compatible severity target that links observable canopy stress signals with fruit-level BER manifestation. Accordingly, the model results demonstrate the feasibility of predicting the combined canopy–fruit RCDI severity classes from UAV vegetation indices, rather than independently predicting fruit-only BER incidence.

### 4.3 Discussion on the MCC-ST semi-supervised learning framework based on consensus mechanism

The MCC-ST framework demonstrates exceptional performance in addressing the characteristic challenges of agricultural remote sensing, namely the high cost of data labeling and the prevalence of vast amounts of unlabeled data. Its core mechanism leverages multi-model consensus and cyclic self-training to effectively resolve the issue of noise accumulation inherent in traditional semi-supervised learning.

Traditional semi-supervised learning methods typically rely on a single classifier to generate pseudo-labels for samples predicted with high confidence. This approach is highly susceptible to confirmation bias, where the model continuously reinforces its own erroneous predictions, leading to an exponential amplification of errors during iterations (Ding et al., 2025). The MCC-ST framework introduces multiple base classifiers with distinct underlying principles. SVM is based on the maximum margin hyperplane theory (Hearst et al., 1998), LR is based on probabilistic statistical models (Zou et al., 2019), and KNN is based on distance metrics in feature space (Taunk et al., 2019). According to ensemble learning theory, the errors of heterogeneous classifiers are often uncorrelated (Mohammed and Kora, 2023), which theoretically satisfies the condition of the independence assumption. Given the relatively strong initial performance of the base classifiers, the initial value of the framework’s signal-to-noise ratio R is large, fulfilling the assumptions made during method construction. Consequently, during the self-loop process, although R exhibits fluctuations (decreasing, increasing, and then decreasing), it overall demonstrates a quasi-exponential upward trend. The ablation experiments in this study confirm that the triple-model collaboration outperforms dual-model combinations, which in turn outperform single-model semi-supervised learning. This demonstrates the critical role of consensus-based collaboration in noise suppression.

Compared to the deep learning-based semi-supervised methods currently popular in agriculture, MCC-ST offers significant advantages in terms of low computational overhead and interpretability. Unlike black-box semi-supervised frameworks based on deep learning (Wu and Prasad, 2018), MCC-ST is supported by an interpretable probabilistic rationale and empirical validation, allowing it to operate effectively in the current small-sample scenario. This strongly demonstrates that in BER monitoring tasks where large-scale labeled data is unavailable, enhancing data quality is more critical than the specific choice of model algorithm.

Although the optimized grid-search parameter-tuning results in **Table 8** demonstrate the best achievable performance under the selected parameter configuration, the repeated random-seed validation provides a more conservative estimate of model stability under limited labeled samples. The confidence intervals and standard deviations in **Table 9** show that MCC-ST + DT and MCC-ST + RF maintained relatively stable within-dataset performance under repeated data partitioning. In contrast, the original DT and RF models showed larger variability and lower mean accuracy, indicating that the small labeled dataset alone was insufficient to support stable disease grading.

Through its consensus mechanism, MCC-ST effectively reduced pseudo-label noise and improved model performance under the current small-sample conditions. Furthermore, unlike co-training methods that rely on multimodal data (Gómez et al., 2021), MCC-ST achieves synergy through model heterogeneity on a single set of vegetation indices. This reduces the dependence on multimodal sensors and suggests the potential of MCC-ST for low-cost agricultural Internet of Things (IoT) applications, although further validation under diverse scenarios is still required.

Nevertheless, the complete independence of base classifiers is difficult to guarantee in practice because all models are trained on the same labeled samples and the same vegetation-index feature set. If the errors of different classifiers are positively correlated, the probability of reaching a consensus on an incorrect pseudo-label may increase, and the theoretical signal-to-noise ratio derived under the independence assumption may be overestimated. Therefore, the independence assumption in this study should be interpreted as an idealized analytical condition. The practical effectiveness of MCC-ST relies not on complete independence, but on sufficient error diversity and complementarity among heterogeneous classifiers. The comparative results with single-model self-training and dual-model co-training indicate that the proposed triple-model consensus mechanism still improves pseudo-label quality under this non-ideal condition.

## 5 Conclusion

Addressing the critical need to safeguard the yield and quality of field crops against abiotic stresses, this study utilized processing tomatoes as a model crop to develop a multi-model self-loop semi-supervised learning framework. By targeting the challenges of label scarcity, lack of targeted prevention, and the labor-intensive nature of large-scale crop monitoring, the specific conclusions are as follows:

The RCDI provides a canopy–fruit synergistic severity target for UAV-based BER monitoring by integrating fruit incidence with canopy phenotypic symptoms, including functional leaf rolling and young-leaf color changes. By leveraging the physiological mechanisms of obstructed nutrient transport (manifested as leaf rolling, L_R_) and tissue necrosis (manifested as leaf color, L_C_), this index overcomes the temporal lag inherent in traditional monitoring, facilitating precise grading of abiotic stress severity.

A core spectral feature combination comprising GRVI, NDVI, and SAVI was identified as optimal for stress monitoring in processing tomatoes. Through a rigorous three-level screening process, these three indices were proven to precisely characterize leaf pigment changes, canopy structural deformation, and soil background noise, respectively. This combination achieved stable within-dataset binary classification performance, with mean accuracies of 96.91%–97.64% across 30 repeated five-fold cross-validations, and clearly outperformed the permuted-label baselines. These results indicate strong spectral separability within the current dataset and demonstrate preliminary potential for automated severity grading based on the RCDI. The MCC-ST framework effectively overcomes the label scarcity bottleneck typical in large-scale precision agriculture by utilizing a heterogeneous consensus mechanism among multiple models. Under 30 random-seed repeated stratified three-fold cross-validations, the final MCC-ST + RF model achieved an accuracy of 85.07% ± 0.42%, with a 95% confidence interval of 84.92%–85.22%, while MCC-ST + DT achieved an accuracy of 85.00% ± 0.31%. Compared with the corresponding original RF and DT models, the repeated-validation accuracies increased by 16.19 and 24.09 percentage points, respectively. This provides preliminary methodological support for precision variable-rate top-dressing in the field, while further validation under diverse environments and management conditions is still required before large-scale application.

However, this study also has certain limitations. First, while RCDI is supported by physiological pathogenesis and showed better compatibility with UAV-derived vegetation indices than conventional DI in the present comparative validation, its weight assignment was still determined using the Analytic Hierarchy Process rather than data-driven optimization. Therefore, the current RCDI should be regarded as a physiologically constrained index rather than a fully optimized empirical index. Future work should incorporate larger multi-year datasets and direct physiological measurements to further optimize the weights and validate the generalizability of RCDI. Although representative physiological measurements supported differences in calcium content and water status between normal plants and BER-symptomatic plants, these measurements did not cover all RCDI sampling points. Future studies should collect paired physiological indicators and symptom data simultaneously at the same sampling points to further validate the physiological mechanism of RCDI. Second, all experiments in this study were conducted at a single site, during one growing season, and using one processing tomato cultivar. Therefore, the robustness and generalizability of the proposed RCDI and MCC-ST framework across different cultivars, growing seasons, soil types, irrigation regimes, and management conditions remain to be further verified. Future work will include multi-year, multi-site, and multi-cultivar experiments to evaluate the transferability and stability of the proposed framework under more diverse agricultural production scenarios.

## Data Availability

The raw data supporting the conclusions of this article will be made available by the authors, without undue reservation.
