# Supplementary material for: UAV-based monitoring of fruit-manifested abiotic stress in crops under label scarcity: a case study of blossom-end rot in processing tomatoes
Source: Front Plant Sci. 2026 Jul 22;17:1882618. doi: 10.3389/fpls.2026.1882618 (PMC13437540; doi:10.3389/fpls.2026.1882618)
Supplement: Supplementary file 1 [file Table1.docx]

Table S1. Summary of sample sets used in different analyses.

| **Analysis** | **Sample source** | **Number of samples** |
| --- | --- | --- |
| Binary BER occurrence classification | Random sampling points | 550 |
| Supervised RCDI-based BER severity grading | Ground RCDI sampling points | 73 |
| MCC-ST labeled training set | Ground RCDI sampling points within each training fold | Approximately two-thirds of the 73 RCDI-labeled samples in each fold |
| MCC-ST severity-unlabeled candidate pool | Random sampling points | 550 |
| MCC-ST pseudo-labeled samples | Generated from the 550 severity-unlabeled random samples | More than 500 under the optimized setting |
| MCC-ST held-out validation set | Ground RCDI sampling points within each validation fold | Approximately one-third of the 73 RCDI-labeled samples in each fold |

Table S2. Hyperparameter search space and optimal hyperparameter values for the DT and RF models.

| **Hyperparameter** | **Grid Search** | **Optimal HyperparametersDT** | **Optimal HyperparametersRF** |
| --- | --- | --- | --- |
| max_depth | 3, 5, 7, 10, None | 5 | 5 |
| min_samples_split | 2, 3, 5, 10 | 2 | 2 |
| min_samples_leaf | 1, 2, 3 | 1 | 1 |
| criterion | gini, entropy | gini | gini |
| n_estimators | 50, 100, 200, 500 | None | 100 |
| max_features | sqrt, log2, None | None | sqrt |

Table S3. Implementation details and key hyperparameter settings of the proposed MCC-ST framework.

| **Hyperparameter** | **Setting used in this study** |
| --- | --- |
| Pseudo-labeling batch size | 20 samples per iteration |
| Pseudo-label acceptance rule | Three-model consensus |
| Base classifiers for pseudo-labeling | LR, SVM, and KNN |
| Final classifiers | DT and RF |
| Training-set update rule | Add accepted pseudo-labeled samples to the training set |
| Stopping criteria | 90% conversion |
| Validation strategy | 30 repeated stratified three-fold cross-validations |
| Machine-learning model | KNN(n_neighbors=5, weights=distance) |
|  | SVM(C=1.0, kernel=rbf, class_weight=balance) |
|  | LR(C=1.0, class_weight=balance, max_iter=1000) |
